# A smartphone-based optical detection for rapid and reliable quantification of bacterial contamination on stainless-steel surfaces

**DOI:** 10.1128/aem.00073-26

**Published:** 2026-04-13

**Authors:** Yuzhen Zhang, Suraj Pathak, Gabriella Curry, Ngoc Vu, Zili Gao, Lili He

**Affiliations:** 1 Department of Food Science, University of Massachusetts 14707, Amherst, Massachusetts, USA; 2 Department of Computer Science, University of Massachusetts 14707, Amherst, Massachusetts, USA; 3 Department of Chemical Engineering, University of Massachusetts 14707, Amherst, Massachusetts, USA; 4 Department of Chemistry, University of Massachusetts 14707, Amherst, Massachusetts, USA; University of Georgia Center for Food Safety, Griffin, Georgia, USA

**Keywords:** smartphone microscope, surface bacterial detection, ATP bioluminescence, 3-mercaptophenylboronic acid, food contact surface, food matrix interference

## Abstract

Surface bacterial monitoring is critical for verifying sanitation effectiveness and preventing cross-contamination in food processing and related industries. However, current methods have notable limitations: culture-based plate counts are time-consuming, and ATP swab tests lack microbial specificity due to interference from non-bacterial ATP. We developed a smartphone-based optical detection method for rapid quantification of bacteria swabbed from surfaces. The method employs 3-mercaptophenylboronic acid (3-MPBA)-coated gold chips to capture bacterial cells, which are visualized using a commercially available $30 smartphone microscope. Automated image processing and quantification are performed using a custom smartphone application based on ImageJ particle analysis. *Salmonella enterica* serovar Enteritidis (SE1045) was used as a model organism for method validation and comparison with the Hygiena UltraSnap ATP monitoring system. The optimized method achieved detection within 5 min and showed a strong linear correlation (*r*² = 0.9947) between surface bacterial load and particle counts over 10⁴–10⁷ cells per 100 cm². The detection limit was 1,978 cells/100 cm², providing approximately twofold greater sensitivity than ATP. Comparable detection performance was observed across multiple bacterial species and strains and across different surface materials, including stainless steel and high-density polyethylene. The method maintained performance in the presence of some common food residues (1% vol/vol or wt/vol), while ATP readings were significantly affected. The 3-MPBA-coated gold chips remained stable for 6 months at room temperature, and the method was validated on a public water fountain surface. Overall, this smartphone-based method provides a rapid, cost-effective optical alternative to conventional ATP-based surface hygiene monitoring tools.

**IMPORTANCE:**

Rapid assessment of surface bacterial contamination is essential for sanitation verification in food processing and environmental hygiene, yet existing tools remain limited by long turnaround times or readouts that cannot distinguish bacterial signals from non-bacterial residues. This study presents a smartphone-based, culture-independent method for quantifying surface-associated bacterial cells, providing a proof-of-concept optical approach for routine hygiene monitoring under defined conditions. The approach enables rapid visualization and enumeration of bacterial contamination while reducing interference from common non-microbial residues compared with ATP-based assays. By demonstrating consistent performance across multiple bacterial species, surface materials, and selected real-world environments, this work supports the utility of low-cost optical tools for surface hygiene monitoring. Overall, the method contributes an accessible framework for improving on-site assessment of bacterial surface contamination in applied microbiology settings.

## INTRODUCTION

Bacterial contamination on environmental surfaces poses serious risks across food processing (1, 2), healthcare (3, 4), and pharmaceuticals (5, 6). Such contamination can lead to foodborne illness, healthcare-associated infections, product recalls, and substantial economic losses (7, 8). Although surface sanitization procedures are routinely applied, continuous surveillance of overall bacterial contamination levels remains essential to verify cleaning effectiveness, guide corrective actions, safeguard product quality, and prevent pathogen transmission (3, 9, 10). While no universal threshold for surface contamination has been established, regulatory agencies require general environmental bacterial monitoring as part of sanitation standard operating procedures in the food industry (11, 12) and recommend routine sampling in healthcare settings to identify potential reservoirs and trace contamination (13).

Currently, aerobic plate count (APC) and ATP bioluminescence swabs are the primary tools for surface monitoring (14, 15). APC is reliable but slow, requiring 3–5 days for results, limiting its use to high-risk sites such as ready-to-eat meat and poultry facilities for highly reliable results (16–18). ATP swabs are faster and widely used for routine hygiene checks, but their reliability is undermined by cross-reactivity with food residues (e.g., proteins, leafy greens, milk, meat), variability across brands of up to three logs for the same species (19), and susceptibility to environmental conditions (20–22). As a result, ATP readings are often treated only as general hygiene indicators, requiring confirmation with APC or polymerase chain reaction (PCR) (23), which increases labor and cost burden. Given these limitations, there remains a strong demand for a rapid and reliable method for detecting bacterial cells on environmental surfaces, enabling timely interventions and improved product quality and safety.

In recent years, smartphone-based detection methods have gained attention for microbiological applications due to their portability, widespread accessibility, and real-time imaging capabilities (24, 25). Because bacterial cells are microscopic and transparent, most smartphone-based approaches rely on autofluorescence (26), fluorescence labeling (27, 28), or colorimetric labeling (29, 30). For example, Buchanan et al. (26) developed an autofluorescence-based system using a 405 nm LED excitation source and an acrylic film filter to directly image bacteria localized within a confined surface area. This approach enables simple, label-free detection when bacterial cells accumulate to sufficiently high local surface densities (on the order of 10⁴ CFU cm⁻²) but is challenging to apply to surfaces where bacterial cells are randomly and sparsely distributed. In addition, autofluorescence signals were shown to be sensitive to background constituents such as BSA, NaCl, and tap water, highlighting challenges associated with matrix interference. Lin et al. (30) reported a smartphone-assisted platform for *Campylobacter coli* detection using loop-mediated isothermal amplification (LAMP), achieving 10-fold higher sensitivity than PCR and successful application in meat samples. Similarly, Xue et al. (31) developed a colorimetric biosensor using MnO_2_ nanozymes to detect *Salmonella* in spiked chicken samples without biomolecular extraction. However, this method required delicate handling and precise color interpretation, limiting field applicability. Rajendran et al. (27) designed a fluorescence nanoparticle-based immunoassay coupled with lateral flow and smartphone readout, though its applicability in food matrices has not been validated.

Despite advances in fluorescence- and microfluidics-based smartphone sensors, several factors limit their broad application for routine surface hygiene monitoring: (i) experimental parameters such as excitation power and exposure time must be carefully optimized to avoid photobleaching or oversaturation (32); (ii) multi-step image processing introduces operator bias and errors (33); (iii) smartphone-to-smartphone variability in camera performance results in inconsistent image quality (34). Beyond these technical considerations, many smartphone-based detection systems have been validated primarily in aqueous bacterial suspensions or localized droplet-deposited samples, rather than in surface swab-based testing, where bacteria are typically dispersed and coexist with food residues and other environmental interferences. Together, these factors highlight the gap between current smartphone-based methods and their broader application for general surface hygiene monitoring, underscoring the need for a simple, reliable, and user-friendly approach for assessing surface-associated bacterial contamination in real-world environments.

Previously, we developed an optical detection method for bacterial cells recovered from stainless-steel surfaces using a low-magnification (10×) objective on a standard light microscope (35, 36). This approach employed 3-mercaptophenylboronic acid (3-MPBA)-coated gold chips, where boronic acid groups form reversible covalent bonds with cis-diol groups in bacterial surface glycans. Because glycans are present on both gram-negative and gram-positive bacteria, 3-MPBA-coated chips enable broad-spectrum, bacteria-selective capture rather than species-specific recognition (37, 38). In addition, the hydrophobic nature of the 3-MPBA-coated surface increases light scattering of the captured bacterial cells by repelling water and reducing water residues on the surfaces, enhancing their visibility under a low-magnification microscope (37). As a result, the method selectively captures bacterial cells while minimizing interference from many non-bacterial residues, enabling reliable detection in complex surface environments. When combined with an optimized swab protocol, surface bacteria could be detected within 2 h, with a limit of detection (LOD) of 7.2 × 10⁴ cells/100 cm². While this method demonstrated promise for industrial applications due to its relatively rapid detection, low cost, and minimal instrumentation, further improvements in speed, simplicity, sensitivity, and cost-efficiency are needed to support broader practical implementation.

The objective of this study was to advance an optical detection method for general bacterial cells recovered from environmental surfaces by integrating it with a smartphone-based platform to improve simplicity and speed and to benchmark its performance against commercial ATP swab tests. *Salmonella enterica* (SE1045), a major foodborne pathogen, was selected as the model bacterium to establish and validate the method, while additional bacterial species and strains were included to evaluate broader applicability. The feasibility of replacing a laboratory microscope with a smartphone-based microscope for bacterial visualization on 3-MPBA-coated gold chips was first assessed. A customized smartphone application was then developed for automated image capture and particle quantification and validated against ImageJ software on a personal computer. The detection protocol was further optimized to reduce procedural complexity and enhance speed. Subsequently, the method’s quantification capability, performance on different surface materials, tolerance to 1% food residues, and room-temperature stability were evaluated and compared with ATP swab tests. Finally, applicability was demonstrated by monitoring bacterial contamination on a public water fountain surface. To our knowledge, this study represents one of the first demonstrations of a smartphone-based optical method for quantifying overall bacterial contamination recovered from surface swabs.

## RESULTS AND DISCUSSION

### Establishment of the simplified optical detection method using a smartphone microscope

**Fig 1:**
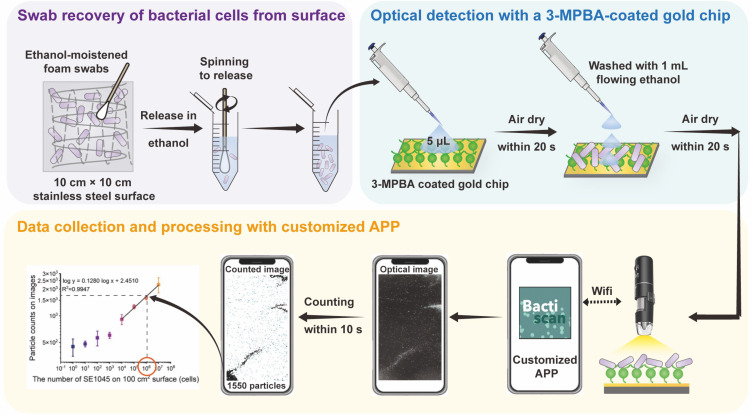
Scheme of steps of the smartphone-based optical detection of bacterial cells loading on stainless-steel surfaces.

To develop a simplified optical detection protocol (Fig. 1), three major modifications were introduced to our previous method (35): (i) replacing the laboratory microscope with a smartphone-based device to improve accessibility and cost-effectiveness; (ii) developing a custom smartphone application to integrate image acquisition and particle counting; and (iii) using ethanol instead of water-based solvents to accelerate drying.

**Fig 2:**
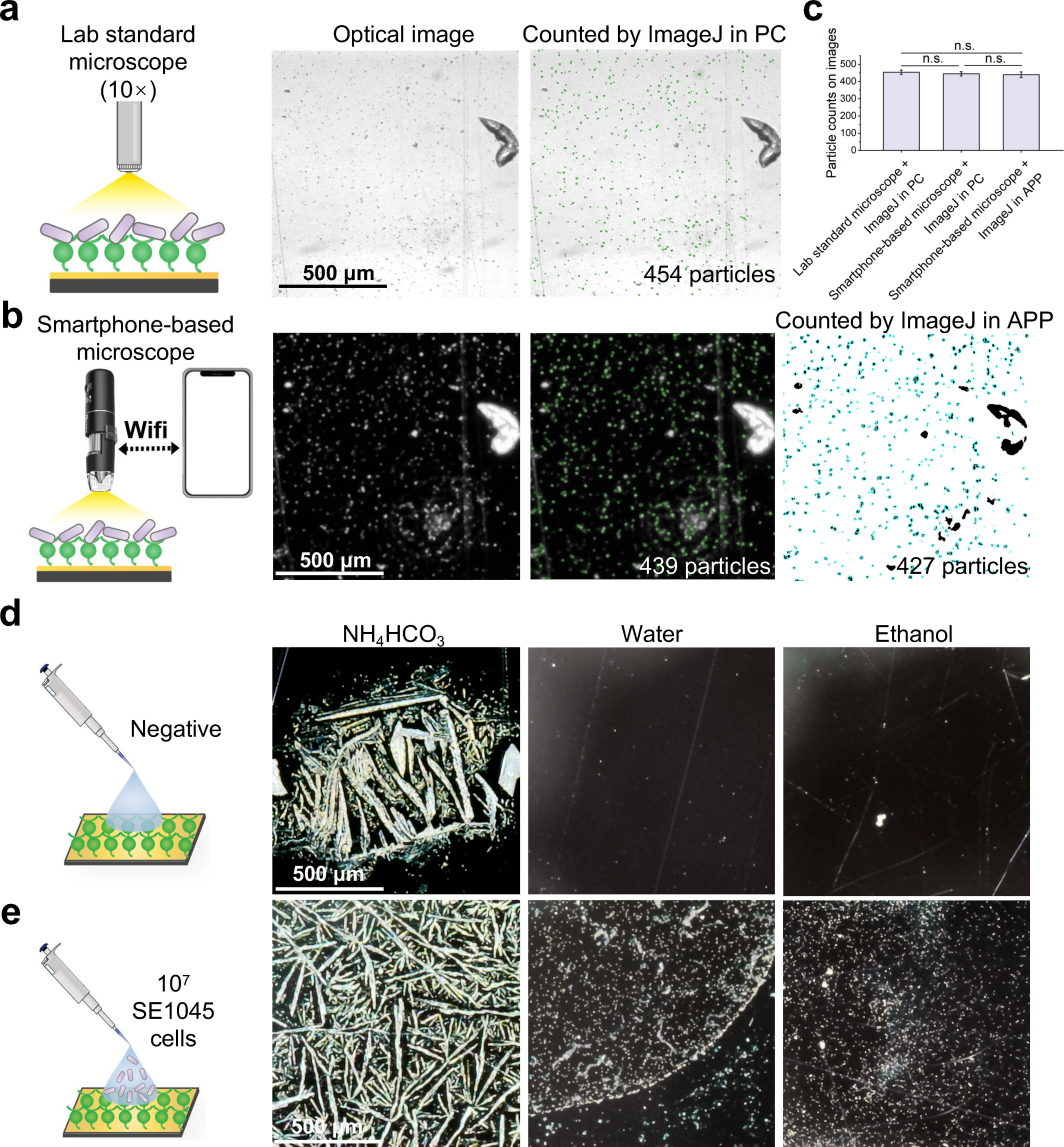
Key factors of the smartphone-based method for bacterial detection. (**a**) Optical image and counted image through ImageJ in PC processing of SE1045 cells on 3-MPBA-coated gold chips under a standard lab optical microscope with a 10× magnification lens; (**b**) optical image and counted images through the ImageJ in PC and ImageJ in a customized smartphone app processing of SE1045 cells on 3-MPBA-coated gold chips under the smartphone-based microscope; (**c**) comparison of the particle counts on images that were processed in panels **a** and **b**; (**d**) optical images (smartphone-based microscope) of 5 μL of NH_4_HCO_3_, water, or ethanol dropped on 3-MPBA-coated gold chips after air drying; (**e**) optical images (smartphone-based microscope) of 5 μL of NH_4_HCO_3_, water, or ethanol with 10^7^ SE1045 cells dropped on 3-MPBA-coated gold chips after air drying. Statistically significant differences in panel (**c**) were determined by paired *t*-test; n.s. illustrated no significant difference level. The scale bar represented 500 μm length. Images shown in this figure were cropped to 40% of the original image area (2 mm × 1.2 mm) for improved visualization. The data presented in panel **c** were obtained by particle counting within the cropped images, not the full original image area. Data are represented as the mean ± standard deviation (SD) of three technical replicates (*n* = 3*).*

The performance of the smartphone microscope was evaluated against a standard laboratory microscope using identical sample preparations (35). Although contrast differed, bacterial particles detected as black dots with the laboratory microscope appeared as white particles at corresponding positions in smartphone images (Fig. 2a and b). Particle counts analyzed by ImageJ on a personal computer (PC) showed no significant differences between the two imaging platforms (Fig. 2c), confirming the smartphone microscope as a viable replacement.

To streamline imaging and analysis, a customized smartphone application was developed that integrates ImageJ with JavaScript-based automation for image acquisition, processing, and quantification (Fig. S1). The app’s particle identification and counting functions were validated against PC-based ImageJ, with no significant differences observed (Fig. 2b and c), confirming accuracy and reliability. The automated pipeline generated results within 10 s of image capture. To ensure consistency across users and locations, key parameters—threshold values, particle size range, and bandpass filtering—were optimized and pre-set, while retaining the option for manual adjustment to accommodate specific samples. The combination of a smartphone microscope with automated processing minimized operator bias and errors from manual adjustments. Compared with other smartphone-based bacterial detection methods relying on autofluorescence or fluorescence labeling, which demand complex reaction control and parameter optimization (24, 32), this approach offered a more straightforward, accessible, and user-friendly alternative.

To evaluate the applicability of the detection method across different smartphone microscope hardware, three commercially available smartphone-based microscopes from different vendors (IWOBAC, Jiusion, and BEBANG) were tested using the same SE1045 sample. As shown in Fig. S2, all three devices enabled visualization of captured bacterial cells as distinct bright dots on the 3-MPBA-coated gold chips. Differences in image appearance were observed among devices, primarily due to variations in optical configuration and illumination conditions. For example, the Jiusion system exhibited a light yellow background, attributable to its yellow LED illumination. Because the effective field-of-view area differed among devices as a result of distinct hardware setups, particle counts were normalized to counts per square millimeter to enable direct comparison (Table S1). After normalization, the coefficient of variation (%CV) across the three smartphone microscope systems was 9.2%, indicating low variability in image acquisition, particle imaging, and counting. These results demonstrated that the optical detection approach is not limited to a single smartphone microscope platform and is adaptable to several commonly available devices.

While NH₄HCO₃ was previously reported as optimal for 3-MPBA-bacteria interactions (37), it crystallized during drying, obscuring bacteria (Fig. 2d and e). To overcome this, water and ethanol were tested as alternative solvents. Both produced clean backgrounds and allowed clear visualization of 10⁷ SE1045 cells on 3-MPBA-coated chips. However, water generated aggregation bands at droplet edges (Fig. 2e), likely due to high surface tension and the coffee-ring effect. In contrast, ethanol spread uniformly, eliminated coffee-ring formation (Fig. 2e), and reduced drying time from 1 h (water) to 20 s, making it the preferred solvent for this method.

During swab recovery, an interference layer was observed on 3-MPBA-coated chips, likely from detached swab fragments, which obscured bacterial particles (Fig. S3a and b). Flushing with 1 mL ethanol effectively removed this layer, restored a clean background, and revealed previously hidden cells, without affecting particle counts even after repeated washes (Fig. S3a through c). Thus, ethanol washing was essential for image clarity and reliable detection. Importantly, bacterial cells were effectively captured by 3-MPBA in ethanol, despite earlier reports identifying NH₄HCO₃ as optimal for boronic acid-diol interactions (37). This may be due to ethanol-induced protein denaturation exposing additional glycan sites and rapid drying minimizing coffee-ring effects. Unlike NH₄HCO₃ protocols requiring 1 h incubation, the ethanol-based droplet-drying method completed detection in 5 min at a consumable cost of around $1.5 per sample. The smartphone microscope (~$30, reusable) further supports the accessibility and cost-effectiveness of this approach.

### Detection performance across multiple bacterial species and strains

To evaluate the applicability of the smartphone-based detection method across diverse bacteria relevant to food and environmental safety, two *Salmonella enterica* strains—serovar Enteritidis ATCC BAA-1045 (SE1045) and subsp. *arizonae* ATCC 13314 (SA13314)—along with two additional bacterial species, *Escherichia coli* ATCC 25922 (*E. coli*) and *Listeria monocytogenes* ATCC 19111 (*L. mono*), were investigated.

**Fig 3:**
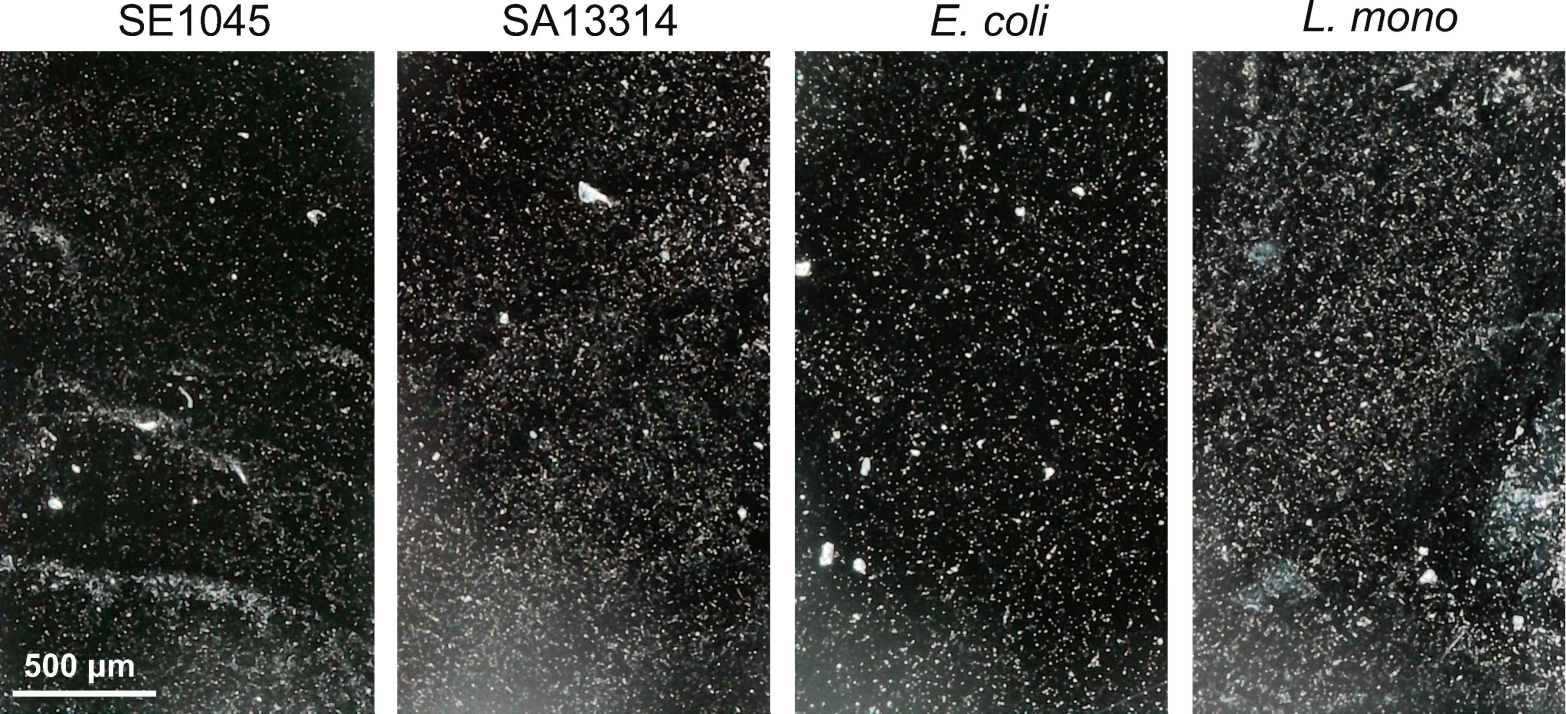
Optical images of bacterial cells captured on 3-MPBA-coated gold chips following swab recovery from stainless-steel surfaces. Four bacterial species were evaluated: *Salmonella enterica* serovar Enteritidis ATCC BAA-1045 (SE1045), *Salmonella enterica* subsp. *arizonae* ATCC 13314 (SA13314), *Escherichia coli* ATCC 25922 (*E. coli*), and *Listeria monocytogenes* ATCC 19111 (*L. mono*). Images were acquired under identical imaging conditions using a smartphone-based microscope. Scale bars represent 500 µm.

Representative optical images of bacterial cells recovered from stainless-steel surfaces and captured on 3-MPBA-coated gold chips are shown in Fig. 3a. Distinct bright dots corresponding to individual bacterial cells were observed for all four organisms under identical imaging conditions, indicating successful capture and visualization across both gram-negative (*Salmonella* spp. and *E. coli*) and gram-positive (*L. mono*) bacteria. Because particle counts observed in the images reflected differences in the initial surface-spiked bacterial levels, detection performance was evaluated by calculating recovery efficiency, which was obtained by back-calculating the background-subtracted particle counts from the imaged field of view and release volume and comparing this value with the corresponding surface-spiked bacterial numbers (Table 1). As summarized in Table 1, comparable recovery efficiencies were obtained across all tested bacteria, with no statistically significant differences observed among species or strains (*P* > 0.05).

**TABLE 1:** Recovery efficiency of different bacterial strains and species from stainless-steel surfaces using 3-MPBA-coated gold chips^*a*^

Bacterial name	Surface spiked bacterial number (CFU/100 cm^2^)	Net particle counts on images	Recovery efficiency (%)
SE1045	(1.37 ± 0.21) × 10^6^	1,360 ± 51.74	41.95 ± 6.35
SA13314	(1.6 ± 0.57) × 10^6^	1,506 ± 88.42	40.36 ± 7.93
*E. coli*	(1.16 ± 0.36) × 10^6^	1,381.67 ± 95.32	50.92 ± 8.22
*L. mono*	(2.26 ± 0.02) × 10^6^	2,532.67 ± 43.00	48.94 ± 11.39

^*a*^ Surface-spiked bacterial levels were determined by plate counting, and net particle counts were obtained from background-subtracted optical images acquired using a smartphone-based microscope. Data are presented as mean ± SD (*n* = 3).

The calculated recovery efficiencies represent the combined effects of swab recovery and capture efficiency on the 3-MPBA-coated gold chips and were therefore not expected to approach 100%. Nevertheless, substantial numbers of bacterial cells were consistently detected across all organisms. These results are consistent with prior reports demonstrating comparable interactions between 3-MPBA and bacterial surface glycans across gram-negative and gram-positive bacteria (37, 38) and support the broad-spectrum applicability of the method for general bacterial contamination assessment on surfaces, with potential relevance to scenarios involving mixed bacterial populations. It should be noted that the recovery efficiencies reported here were obtained using surface-spiked bacterial levels on the order of 10⁶ cells, representing an intermediate contamination level. Based on our previous observations, the capture efficiency of 3-MPBA-coated gold chips tends to increase as bacterial surface loading decreases (36). This behavior may be attributed to saturation of available binding sites on the gold surface at higher bacterial loads, whereas at lower bacterial levels, a larger fraction of the total cells present may be captured, suggesting that recovery efficiency may be higher at lower contamination levels and may decrease at substantially higher bacterial loads.

### Quantitative performance of smartphone-based detection and its comparison with the ATP swab tests

**Fig 4:**
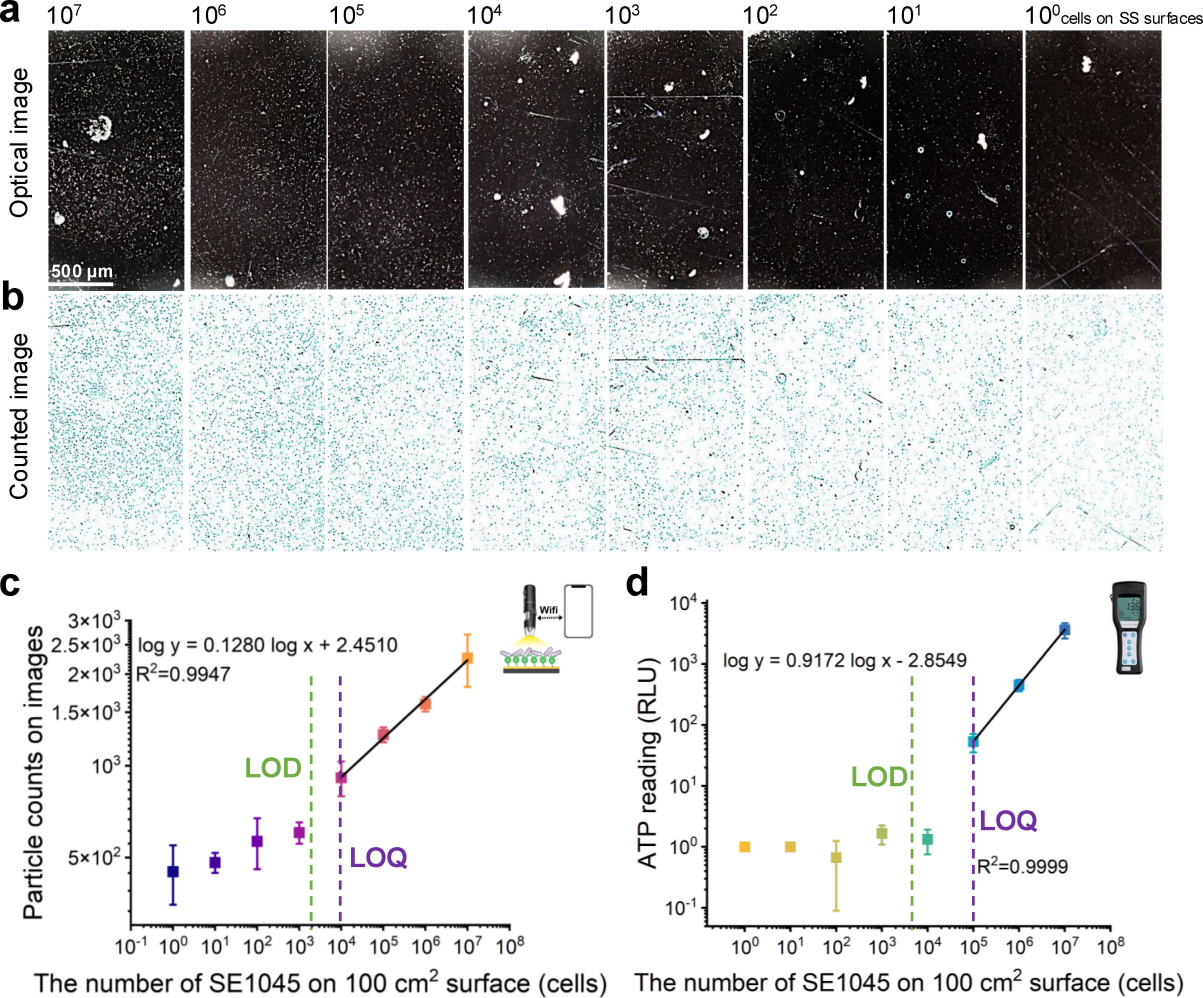
Quantification capability of the smartphone-based method for detecting SE1045 cells on stainless-steel surfaces and its comparison with the ATP swab tests. (**a**) Optical images (smartphone-based microscope) of SE1045 cells on 3-MPBA-coated gold chips recovered from stainless-steel surfaces with different SE1045 cell numbers (from 10^7^ to 10^0^ cells per surface); (**b**) counted images from optical images in panel **a** through the processing of customized smartphone app; linear correlation between the number of SE1045 cells on 10 × 10 cm^2^ stainless-steel surfaces and (**c**) the particle counts on images in panel **b**; (**d**) the ATP reading results. The scale bar represented 500 μm length. Data are represented as the mean ± SD of three technical replicates (*n* = 3).

Following the establishment of the smartphone-based optical detection method, its quantitative capability for detecting bacterial cells on stainless-steel surfaces was evaluated. The method was applied to 10 × 10 cm² stainless-steel surfaces inoculated with different numbers of SE1045 cells ranging from 10^0^ to 10^7^ cells. As shown in Fig. 4a, the number of white dots on the 3-MPBA-coated gold chips progressively decreased as the number of SE1045 cells on the surfaces decreased. While some larger bright clusters were observed, likely originating from airborne dust particles or swab fragments, these were automatically excluded from particle counting (Fig. 4b, marked with blue solid lines) based on size and threshold settings within the image analysis parameters. A strong linear correlation (*r*² = 0.9947) was observed between particle counts and surface-spiked cell numbers (Fig. 4c). The LOD was calculated based on a negative-control surface without bacterial inoculation that was swabbed and processed through the same workflow, resulting in an LOD of 1,978 cells/100 cm². The limit of quantification (LOQ) was defined as the lowest surface-spiked level that showed a statistically significant difference (*P* < 0.001) compared with lower concentrations and was determined to be 10⁴ cells/100 cm². These LOD and LOQ values demonstrate the method’s sensitivity and quantitative performance. However, they represent analytical detection limits under the defined swab-based protocol and should not be interpreted as universal field detection limits, as surface recovery efficiency and sampling conditions may vary in practical applications.

Compared to the previous optical detection method (35), this approach exhibited a 1.5-log lower LOD, indicating an improvement in sensitivity and detection capability. The improved sensitivity may be attributed to the ethanol-based drying process, which likely enhanced bacterial adhesion to the 3-MPBA-coated gold chips compared to random collisions in an aqueous environment. Buchanan et al. (26) developed a smartphone-based autofluorescence imaging method for detecting bacteria on laboratory surfaces without swab sampling, achieving an LOD of 10⁴ cells/cm². However, this value was derived from microliter-scale droplet deposition and area-based conversion rather than swab-based recovery of dispersed surface contamination. Although the reported units appear similar, the two approaches are therefore not directly comparable. In contrast, the swab-based sampling strategy used here concentrates bacteria from a larger surface area, enabling effective detection of low-level, widely distributed bacterial contamination relevant to surface hygiene monitoring.

For comparison, the Hygiena UltraSnap ATP monitoring system was tested on stainless-steel surfaces inoculated with varying concentrations of SE1045 cells. The relative light units (RLU) exhibited a strong linear correlation (*r*² = 0.9999) with bacterial counts in the range of 10⁵–10⁷ cells (Fig. 4d). However, ATP readings below 10⁴ cells showed no significant differences, whereas values at 10⁵ cells were significantly higher (*P* < 0.001), establishing the LOQ at 10⁵ cells/100 cm². The LOD was calculated as 4,110 cells/100 cm². This detection limit was approximately twofold higher than that of the smartphone-based method, underscoring the superior quantification capability of the latter. It should be noted that the LOD of ATP swab tests can vary considerably depending on the brand, bacterial strain, and testing conditions. For example, Omidbakhsh et al. (19) reported LOD values ranging from 10² to 10⁵ CFU among four commercial ATP systems tested against *Staphylococcus aureus*. Furthermore, the sensitivity of ATP swabs has been shown to differ between gram-positive and gram-negative bacteria due to variations in cell lysis efficiency (39). The present results, therefore, apply specifically to the Hygiena UltraSnap ATP monitoring system and SE1045 cells, and the sensitivity may differ when other bacterial species are tested.

**Fig 5:**
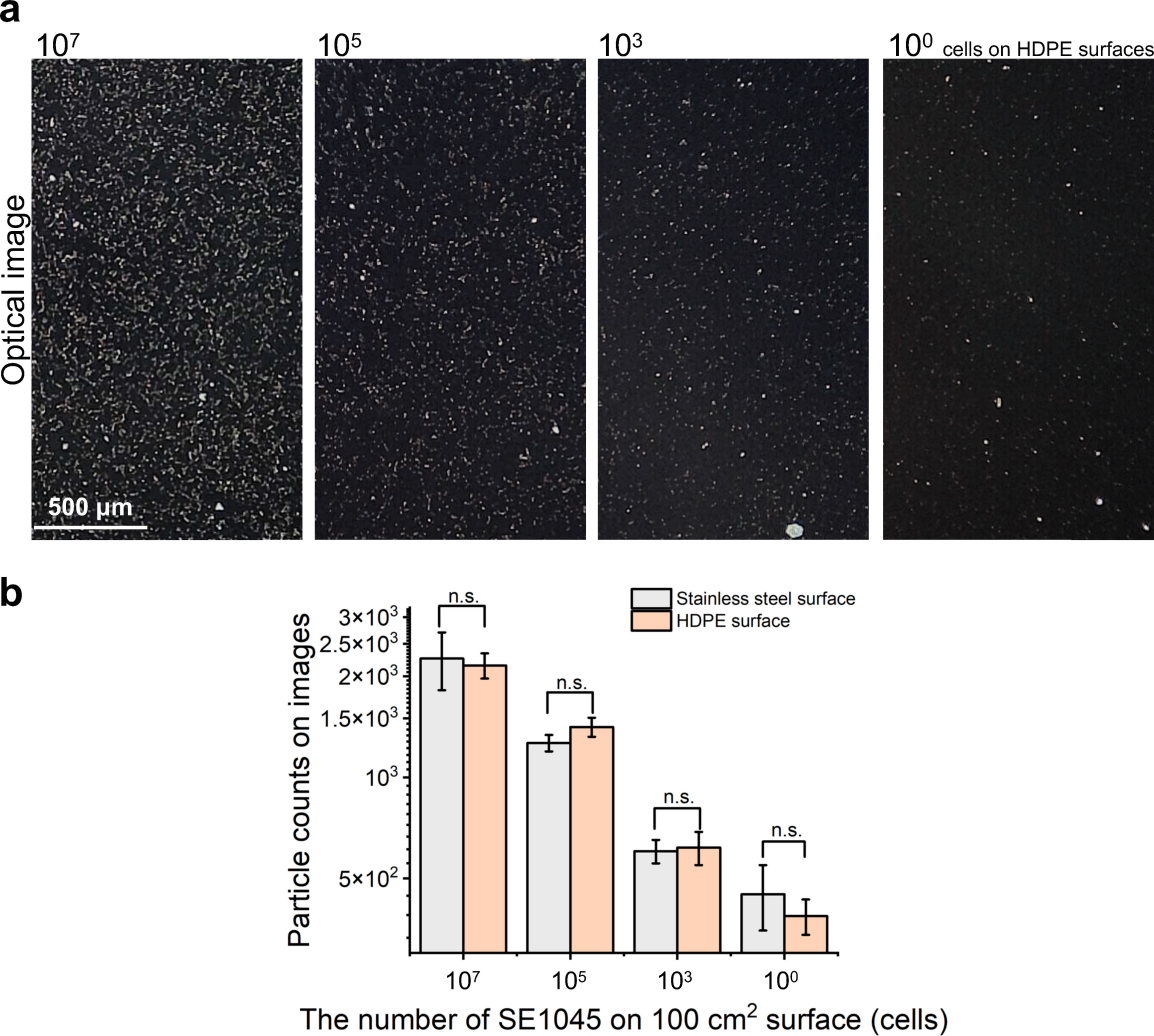
Performance of the smartphone-based optical detection method across different surface materials. (**a**) Representative optical images of SE1045 captured on 3-MPBA-coated gold chips following swab recovery from HDPE surfaces inoculated with decreasing bacterial loads (10⁷, 10⁵, 10³, and 10^0^ cells per 100 cm²); (**b**) quantitative comparison of particle counts obtained from stainless-steel and HDPE surfaces across the same range of surface-spiked bacterial levels. Bars represent the mean ± standard deviation from three technical replicate measurements. Statistical comparisons between surface types at each bacterial level were performed, with “n.s.” indicating no statistically significant difference (*P* > 0.05). Scale bars represent 500 µm.

Because different surface materials are commonly encountered in real-world food and environmental settings, high-density polyethylene (HDPE) was evaluated as an additional surface material to assess the detection capability of the smartphone-based method. SE1045 cells were surface-spiked onto HDPE at varying bacterial levels and recovered by swabbing prior to optical analysis. As shown in Fig. 5a, bacterial cells recovered from HDPE surfaces were clearly visualized as distinct bright features, with particle density decreasing in accordance with reduced surface-spiked bacterial numbers. Quantitative comparison of particle counts obtained from stainless-steel and HDPE surfaces across four bacterial loading levels showed no statistically significant differences (Fig. 5b), indicating comparable detection performance on these two surface materials. Although surface material has been reported to influence swab recovery efficiency (40), the present results suggest that stainless-steel and HDPE surfaces did not markedly affect detection outcomes under the conditions tested. Evaluation of additional surface types, such as glass, polystyrene, silicon, and rubber, will be necessary to further assess the influence of surface properties on swab recovery and detection performance.

### Bacterial detection performance with food matrices using the smartphone-based detection and its comparison with the ATP swab tests

**Fig 6:**
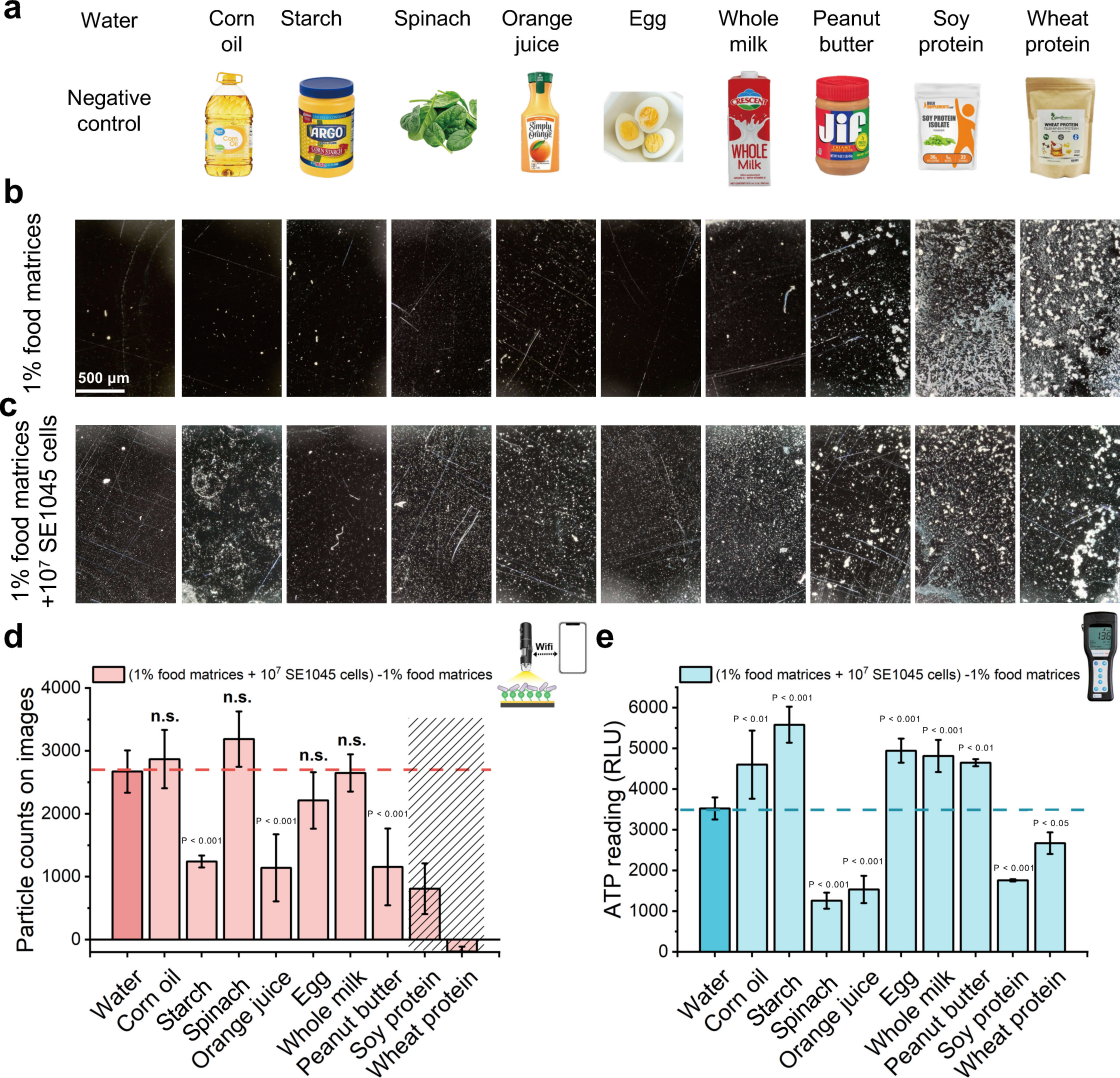
Evaluation of food matrices’ influence on the detection performance of the smartphone-based method and its comparison with the ATP swab tests. (**a**) Pictures of food matrices that were introduced to investigate their influence on the background and bacterial detection performance of the smartphone-based method and ATP swab tests; (**b**) optical images (smartphone-based microscope) of samples recovered from surfaces with 1% different food matrices on 3-MPBA-coated gold chips; (**c**) optical images (smartphone-based microscope) of samples recovered from surfaces with 1% different food matrices and 10^7^ SE1045 cells on 3-MPBA-coated gold chips; (**d**) comparison of particle counts with the presence of different food matrices, which were obtained by subtracting the counts in panel **b** from panel **c**. The pink dashed line was drawn based on the average value of the darker pink bar to illustrate the control value. The diagonal pattern that covered the results from soy and wheat protein represented the inaccurately calculated results due to big trunks on the images; (**e**) comparison of ATP reading results with the presence of different food matrices, which were obtained by subtracting the results of only 1% food matrices on surfaces from 1% food matrices and 10^7^ SE1045 cells on surfaces. The blue dashed line was drawn based on the average value of the darker blue bar to illustrate the control value. Statistically significant differences were determined by paired *t*-tests, *P*-values were marked on figures, and n.s. illustrated no significant difference level. The scale bar represented 500 μm length. Data are represented as the mean ± SD of three technical replicates (*n* = 3).

In food processing environments, residual food matrices may interfere with bacterial detection (41). To evaluate matrix effects, nine representative foods—corn oil, starch, spinach, orange juice, egg, whole milk, peanut butter, soy protein, and wheat protein—were prepared as 1% (vol/vol or wt/vol) solutions (Fig. 6a; Fig. S4a).

To evaluate background interference, stainless-steel surfaces were distributed with 1% food matrices and analyzed using the smartphone-based method. Soy protein and wheat protein caused the greatest interference, covering 3-MPBA-coated chips with irregular particles of varying sizes (Fig. 6b; Fig. S4b). Peanut butter also introduced background noise, likely from fragmented residues, while orange juice generated large particulates that were excluded by the predefined size thresholds. Corn oil, starch, egg, and whole milk produced clean backgrounds comparable to the water control (Fig. S5a). However, spinach produced elevated particle counts, likely from fragmented plant tissue and/or intrinsic bacterial contamination. Further validation is necessary to confirm the composition of these background particles in future studies. Quantitatively, corn oil, starch, orange juice, egg, and whole milk produced background signals consistent with the negative control. By contrast, ATP readings increased significantly for all matrices (*P* < 0.01), with particularly high signals for spinach and orange juice (*P* < 0.001; Fig. S5b), consistent with previous reports of strong organic-matter interference in ATP tests (21, 42).

To assess the impact of food matrices on bacterial detection, a spike-recovery approach was used. Surfaces were treated with 1% matrices spiked with 10^7^ SE1045 cells and analyzed by the smartphone method and ATP swabs. Except for soy and wheat proteins—which impaired particle identification—the smartphone method showed higher particle counts than matrix-only backgrounds for most foods (Fig. 6c; Fig. S4c). For corn oil, egg, and whole milk, added particles were attributable to bacteria, as these matrices did not generate background. Compared with the bacterial control (Fig. S5a), particle counts were reduced in the presence of starch, likely due to its strong adsorption capacity sequestering cells before reaching the 3-MPBA-coated chip. Orange juice also yielded lower detection, attributable to its acidic environment, which disfavors boronic acid-diol interactions that are enhanced under basic conditions (43). Peanut butter produced large aggregates that reduced effective chip area for cell capture. Because soy and wheat proteins severely biased counting, these were shaded and excluded from direct statistical comparison (Fig. 6d; Fig. S5a).

To isolate bacterial signals, matrix-only backgrounds were subtracted from matrix + cells measurements. Subtracted counts for corn oil, spinach, egg, and whole milk showed no significant difference from control (Fig. 6d). Notably, although spinach elevated background, its subtracted counts matched control, indicating no adverse impact on quantification once background is accounted for. These results indicate that the method maintains stable performance in lipid- and carbohydrate-containing residues (e.g., corn oil, spinach, egg, and whole milk), whereas protein-rich matrices such as soy and wheat proteins substantially interfere with particle visualization and counting. This interference likely arises from residual protein deposition on the chip surface, which obscures bacterial signals and limits reliable quantification, indicating that the current protocol is not suitable for surfaces heavily contaminated with protein-rich residues. Nevertheless, compared with previously reported smartphone approaches, the present optical method demonstrated improved specificity in distinguishing bacterial cell signals from non-bacterial residues across multiple matrices. For example, an autofluorescence-based method generated signals from BSA, NaCl, and tap water that were indistinguishable from bacteria (26). Other smartphone-based methods demonstrated bacterial detection in single matrices such as chicken (31), meat (30), or milk (44), but their performance across a broader range of food residues has not been evaluated.

For ATP swab tests, both non-subtracted and background-subtracted readings remained significantly affected by all matrices (Fig. 6e; Fig. S5b), aligning with reports of limited ATP reliability in the presence of organic residues (39, 45). Direct comparison of the significance analyses in Fig. 6d and e indicated the smartphone method is less susceptible to matrix variability than ATP. Consistently, percent bias (%Bias) and %CV relative to the water control for each food matrix were summarized in Table S2, demonstrating the lower bias and improved precision of the optical detection method for several common food matrices, particularly corn oil, spinach, egg, and whole milk. However, both the optical method and ATP monitoring showed poor performance for protein-rich residues, characterized by high bias and variability.

**Fig 7:**
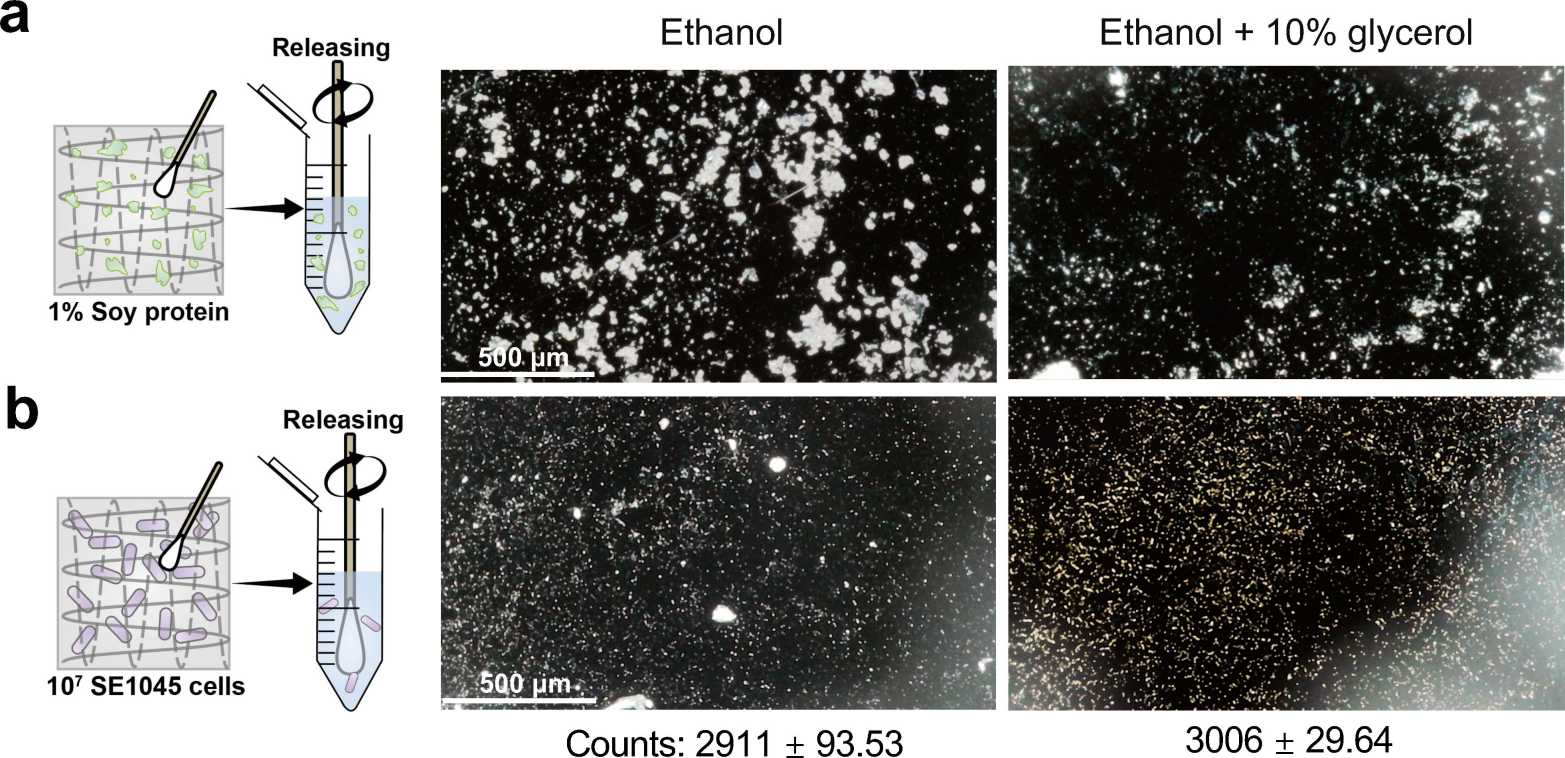
Effects of different release solutions on the capture of proteins and bacteria by 3-MPBA-coated gold chips. Optical images (smartphone-based microscopy) of (**a**) 1% soy proteins and (**b**) 10^7^ SE1045 cells recovered from stainless-steel surfaces and released into ethanol and ethanol with 10% glycerol on 3-MPBA-coated gold chips. The scale bar represented 500 μm length. Data are represented as the mean ± SD of three technical replicates (*n* = 3).

Protein-based foods presented the greatest challenge to the smartphone method, likely due to (1) ethanol-induced protein denaturation/aggregation depositing on the chip surface and (2) exposure of hydrophobic residues that promote adsorption to 3-MPBA (46). To address this, 10% glycerol was added to ethanol to increase solvent hydrophilicity while maintaining rapid drying. Swabbed soy protein residues (Fig. 7a) or SE1045 cells (Fig. 7b) were released into ethanol-glycerol or ethanol alone (control) and deposited onto 3-MPBA-coated chips. Although glycerol markedly reduced protein interference, residual aggregates and streak-like deposits were still observed for soy protein residues (Fig. 7a). For such environments, pre-cleaning steps to remove residual proteins or avoidance of testing prior to routine sanitation are recommended to ensure reliable measurements. Importantly, glycerol addition did not affect bacterial particle counts for SE1045 (Fig. 7b), suggesting that solvent optimization can reduce matrix interference without compromising detection. Further optimization of solvent composition may improve performance in protein-rich environments. The strong retention of protein residues on the chip surface also suggests potential applicability of this platform for food allergen detection.

### Reproducibility and stability of 3-MPBA-coated gold chips across storage duration and operators

In addition to bacterial detection performance, the shelf life of the 3-MPBA-coated gold chips was evaluated, as storage conditions and duration are critical factors for their applicability and reproducibility in real-world industries. The prepared 3-MPBA-coated gold chips were stored at room temperature (26°C) for different time intervals (from 0 day to 6 months) and subsequently tested for background cleanliness (negative control) and bacterial capture capability (positive control) (Fig. S6).

**Fig 8:**
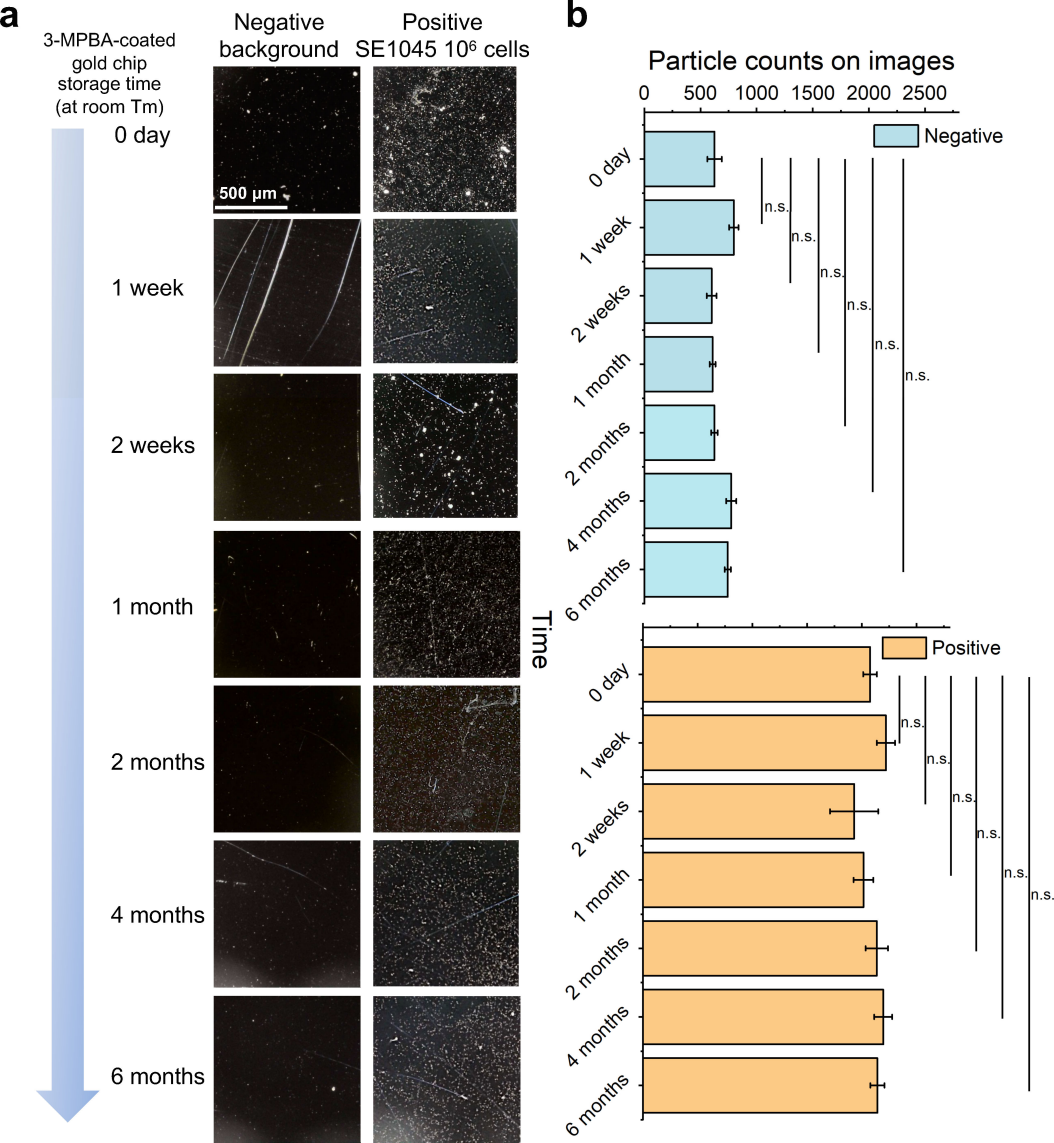
Evaluation of the shelf life of the 3-MPBA-coated gold chips for bacterial detection. (**a**) Optical images (smartphone-based microscope) of the 3-MPBA coated gold chips stored under room temperature for 1 week, 2 weeks, 1 month, 2 months, 4 months, and 6 months to capture nothing (Negative) and 10^6^ SE1045 cells (Positive); (**b**) the bar graphs of particle counts along the storage time on optical images in panel **a**. The blue bars represented the particle counts on negative images, while the orange bars represented the particle counts on images with 10^6^ SE1045 cells. Statistically significant differences in panel **b** were determined by paired *t*-test; n.s. illustrated no significant difference level (*P* > 0.05). The scale bar represented 500 μm length. Images shown in this figure were cropped to 40% of the original image area (2 mm × 1.2 mm) for improved visualization. However, the data presented in panel **b** were calculated using the full original image size. Data are represented as the mean ± SD of three technical replicates (*n* = 3*).*

Over time, images of negative control chips consistently exhibited clean backgrounds, while a consistent density of bacterial cells remained visible on the 3-MPBA-coated gold chips in positive control samples (Fig. 8a). Notably, particle count analysis revealed no significant differences in either negative or positive samples across different storage durations (Fig. 8b). To further quantify stability, %CV was calculated across time points (Table 2), yielding values of 12.60% for negative controls and 4.90% for positive controls. The low %CV observed for positive controls indicates preserved bacterial capture capability during room-temperature storage. The slightly higher %CV observed for negative controls is likely attributable to minor background variability introduced during manual glass slide cutting, which can result in variable surface scratches and background cleanliness. This variability is expected to be reduced through standardized fabrication using automated manufacturing processes. In comparison, according to the manufacturer’s instructions, the ATP swab test used in this study has a maximum storage time of only 1 month at room temperature and 1 year under refrigeration. The extended stability of the 3-MPBA-coated gold chips at room temperature supports their practical suitability for storage under typical laboratory and industrial conditions.

**TABLE 2:** Storage stability of 3-MPBA-coated gold chips evaluated using negative and positive controls^*a*^

Storage time	Negative control (particles/image)	Positive control (particles/image)
0 day	625.33 ± 64	2,075 ± 63
1 week	796.33 ± 41.58	2,220.33 ± 83.36
2 weeks	599.33 ± 44.01	1,929.33 ± 219.98
1 month	607.67 ± 26.16	2,014.67 ± 90.05
2 months	624.33 ± 28.53	2,136.67 ± 102.27
4 months	774.67 ± 44.76	2,195.33 ± 82.28
6 months	743 ± 27.40	2,142 ± 64.97
%CV	12.60	4.90

^*a*^ Particle counts were obtained from optical images acquired using a smartphone-based microscope. Data are presented as mean ± SD (*n* = 3). Overall coefficient of variation (%CV) was calculated across storage time points.

To evaluate inter-operator reproducibility, an undergraduate student with no prior laboratory experience was trained by observing the procedure performed by a trained operator and then independently conducted the detection workflow. Surfaces inoculated with 10⁷, 10⁶, and 0 SE1045 cells were tested. As shown in Fig. S7, particle counts obtained by the trainee were not significantly different from those recorded by the trained operator (*P* > 0.05). To complement significance testing, %CVs were calculated across operators in Table 3, yielding values ranging from 3.67% to 5.91%, which indicate low inter-operator variability and consistent measurement precision. Future studies will include a larger group of non-trained users and prototype-level testing to further evaluate user-to-user variability under real-world conditions.

**TABLE 3:** Inter-operator reproducibility of the smartphone-based detection method^*a*^

Surface load (cells/100 cm²)	Trainer (particles/image)	Trainee (particles/image)	%CV
0	513.25 ± 94.29	485.33 ± 252.95	3.95
10⁶	1,663 ± 88.30	1,578.75 ± 141.68	3.67
10⁷	2,325.75 ± 442.56	2,139 ± 545.01	5.91

^*a*^ Particle counts were obtained by a trained operator and an untrained trainee for surfaces inoculated with different levels of SE1045 cells. Data are presented as mean ± SD (*n* = 3). Coefficient of variation (%CV) was calculated to assess inter-operator reproducibility.

### Real-world application of smartphone-based optical detection on the public water fountain surface

**Fig 9:**
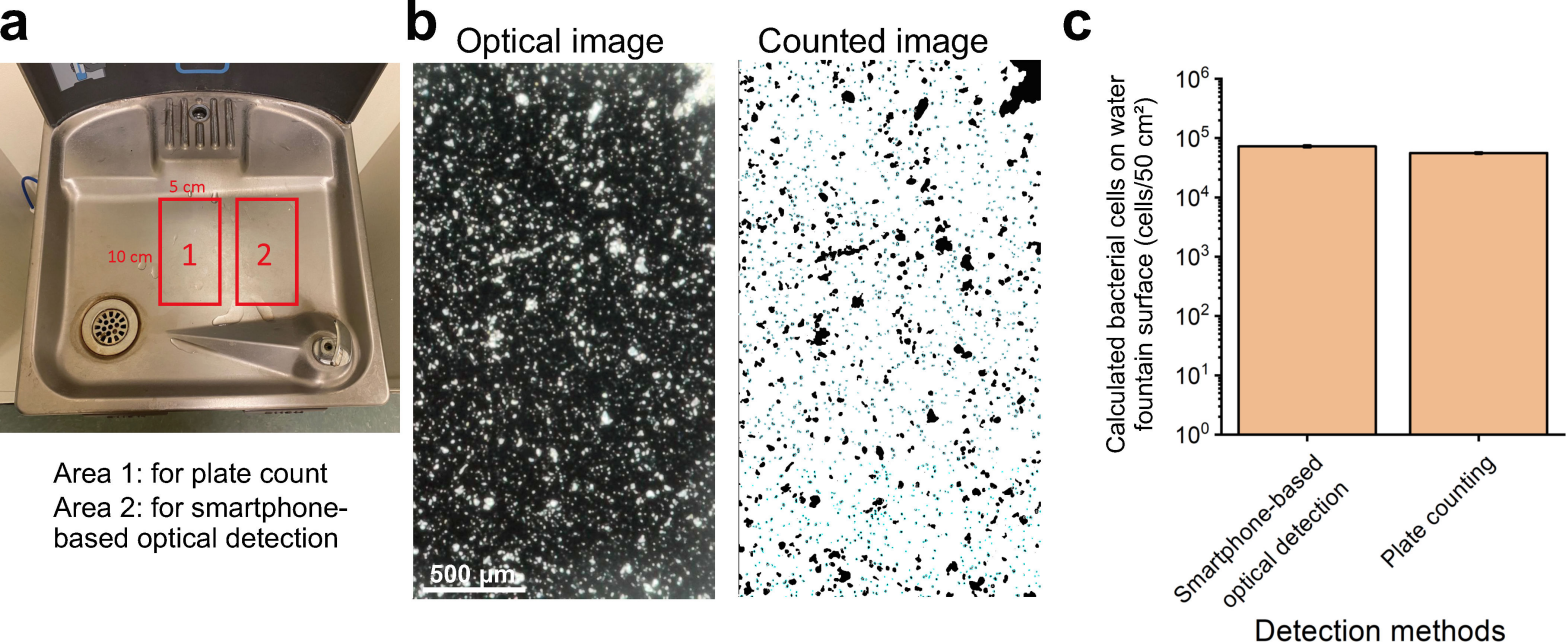
Application of smartphone-based optical detection for assessing bacterial contamination on a public water fountain. (**a**) Photograph of the water fountain showing two swabbing areas: Area 1 was used for plate counting, and Area 2 for smartphone-based optical detection. Each swabbed area measured 5 cm × 10 cm; (**b**) representative optical image (left) acquired using a smartphone-based microscope and the corresponding counted image (right) generated from particles captured on 3-MPBA-coated gold chips from Area 2; (**c**) bar graph comparing calculated bacterial cell numbers on a water fountain surface obtained by smartphone-based optical detection and traditional plate counting. For the optical method, image-based particle counts were converted to surface bacterial levels using the standard calibration curve shown in Fig. 4c. For plate counting, CFU enumerated on tryptic soy agar (TSA) agar were converted using the corresponding standard curve shown in Fig. S8. Data represent the mean ± standard deviation of three technical replicates (*n* = 3). The scale bar represented 500 μm length.

In real environments, bacterial adhesion varies with exposure time and may progress to biofilm formation, which can affect recovery and detection efficiency (47, 48). To demonstrate preliminary real-world applicability, bacterial contamination was assessed on a public water fountain surface in our laboratory building (Fig. 9a). This surface was neither artificially inoculated nor regularly sanitized, allowing natural accumulation of bacterial cells and potential early-stage biofilms, thereby simulating realistic monitoring conditions.

Two adjacent stainless-steel areas (each 5 cm × 10 cm) were swabbed: one was analyzed using the smartphone-based method with 3-MPBA-coated chips, while the other was evaluated by traditional plate counting (Fig. 9a). Optical imaging revealed numerous white particles, including small dots likely corresponding to individual bacterial cells and larger aggregates potentially representing residual debris or biofilm-like structures (Fig. 9b). For quantitative analysis, only particles between 3 and 60 pixels in size—corresponding to single bacterial cells—were included. Based on the optical calibration curve (Fig. 4c), the bacterial load was calculated to be 72,772 ± 2,806 cells, while plate counting yielded 56,073 ± 1,726 cells, calculated using the standard curve shown in Fig. S8 (Fig. 9c). The slightly higher bacterial counts observed using the smartphone-based method may be attributed to two factors: (i) differences in microbial distribution between the adjacent swabbed areas, which could naturally result in sampling variability; and (ii) the inherent distinction between detection principles—plate counting quantifies only viable and culturable cells, whereas the optical method detects all captured bacterial cells regardless of their viability. Some bacterial cells may have been injured, stressed, or inactivated and thus unable to form colonies but remained detectable by the optical system. Despite this, both methods produced results of the same order of magnitude, validating the reliability of the smartphone-based approach. These findings demonstrate that the smartphone-based method can effectively detect bacterial contamination under uncontrolled, real-world conditions, supporting its feasibility as a rapid optical tool for environmental hygiene monitoring.

The sampled fountain surface also exhibited scratches and niches from prolonged use, features known to enhance bacterial adhesion and biofilm formation by providing additional contact points, thereby complicating cleaning and detection (49). As such, the actual bacterial load on the water fountain surface may have been higher than what was detected. While the water fountain material—stainless steel—matches the laboratory surfaces used in this study, further validation is required on other materials, as detection efficiency can vary with surface properties (50). In contrast, Keeratipibul et al. (51) reported that swab recovery did not significantly differ across materials of varying roughness and hydrophobicity, with swab type and surface condition (wet vs dry) being the dominant factors. These contrasting findings highlight the complexity of real-world surfaces, where material type, roughness, hydrophobicity, and micro-niches interact. Thus, additional field validations across diverse surface types and conditions, alongside comparisons with plate counting and ATP swabs, are necessary to fully assess the strengths and limitations of the smartphone-based method.

For SE1045 cells on stainless-steel surfaces, the smartphone-based method outperformed the Hygiena UltraSnap ATP system in quantification, compatibility with food matrices, storage stability, and device cost, while maintaining comparable detection speed (Table 4). Although the method utilizes gold-coated glass slides that are currently disposable, each test requires only a small area of the coated surface, resulting in a relatively low per-test material cost. When accounting for the cost of swabs and reagents used with the 3-MPBA-coated gold chips, the estimated consumable cost per test remains lower than that of commercial ATP swab assays, with additional cost reductions anticipated upon manufacturing scale-up. Future efforts to explore reuse or recycling of the gold coating may further reduce overall costs. Unlike the ATP monitoring system, which relies on fluorescent readings, the smartphone-based method enables direct visualization and enumeration of bacterial cells, enhancing standardization and cross-comparability of results across diverse environments. Compared with smartphone-based fluorescence or colorimetric assays that require precise reaction control and complex image analysis, the optical imaging workflow is simplified through direct light microscopy and automated particle counting, making it user-friendly and adaptable to different skill levels. Its accessibility also supports applications ranging from industrial facilities to resource-limited settings.

**TABLE 4:** Comparison of features of commercially available ATP monitoring system and smartphone-based optical detection

	Hygiena UltraSnap surface ATP monitoring system	Smartphone-based optical detection
Time duration	5 min	5 min
Limit of quantification (cells/100 cm^2^)	10^5^	10^4^
Limit of detection (cells/100 cm^2^)	4,110	1,978
Device cost	$1,800 (ATP meter)	$30 (smartphone microscope)
Consumable cost	$2.6/test	$1.5/test
Applicability with interference of 1% food matrices	Significantly influenced by each food matrix	Unaffected by corn oil, spinach, egg, and whole milk
Storage time (at room Tm)	1 month	at least 6 months

Despite these advantages, the method demonstrated reliable performance only under the controlled laboratory and limited field conditions evaluated in this study. Broader validation across diverse environmental settings and comprehensive workflow reproducibility testing will be necessary before generalizing its reliability for routine field implementation. In addition, the present comparison was limited to a single ATP brand and a defined set of bacterial species. Further evaluation is needed to account for variability across ATP systems and more diverse food matrices, including poultry, seafood, and sanitizer residues. Although ethanol with 10% glycerol reduced protein interference, further solvent optimization is needed. Field studies in pilot plants, dining facilities, and healthcare settings, with direct comparison to plate counting and ATP tests, will strengthen applicability. To improve accuracy, machine-learning-based particle classification could enhance bacterial discrimination and repeatability. Finally, chemical modification of gold chips with selective binding elements may enable species-specific detection of foodborne pathogens, expanding the platform’s utility for contamination monitoring and risk assessment.

### Conclusion

This study developed a smartphone-based optical method for detecting bacterial cells on stainless-steel surfaces, integrating a custom mobile application for automated image processing. The app, embedding ImageJ’s particle-counting function with tailored scripts, enabled real-time quantification consistent with PC-based ImageJ. Ethanol was identified as an effective wetting and releasing solution, enabling uniform bacterial distribution and rapid drying. Using a low-cost (~$30) smartphone microscope and consumables costing approximately $1.5 per test, the method achieved detection within 5 min and demonstrated comparable detection performance across multiple bacterial species and strains. The method exhibited strong quantification capability (*r*² = 0.9947) and an LOD of 1,978 SE1045 cells/100 cm²—representing a twofold lower LOD than the Hygiena UltraSnap ATP system. Comparable detection and quantification performance was also observed on both stainless-steel and HDPE surfaces. Detection was unaffected by 1% corn oil, spinach, egg, or whole milk but was variably influenced by starch, orange juice, peanut butter, soy protein, and wheat protein. In contrast, ATP readings were significantly impacted by all matrices, underscoring the higher specificity of 3-MPBA-coated gold chips toward bacterial cells relative to non-bacterial residues. Ethanol with 10% glycerol further reduced protein interference without compromising detection. Chips remained stable for at least 6 months at room temperature, six times longer than ATP swabs. Applied to a public water fountain surface, the method successfully detected natural bacterial contamination, validating its real-world utility. Overall, the platform offers comparable speed to ATP tests with improved analytical sensitivity and reduced matrix susceptibility under the tested conditions, suggesting potential for commercial implementation. Future work will extend validations across diverse environments and optimize the system for product development.

## MATERIALS AND METHODS

### Bacterial cell preparation

*Salmonella enterica* subsp. enterica serovar Enteritidis (ATCC BAA-1045) (SE1045) was selected as representative bacterium throughout this study. Frozen bacterial cultures were streaked onto TSA (Becton, Dickinson and Company, Difco, Franklin Lakes, NJ, USA) and incubated at 37°C for 16 h. A single colony was then transferred into 10 mL of tryptic soy broth (TSB) (Becton, Dickinson and Company, Difco, Franklin Lakes, NJ, USA) and incubated at 37°C with shaking at 250 rpm for 16 h until reaching the stationary phase. To prepare the bacterial suspension, 1 mL of the incubated culture was centrifuged at 10,000 × *g* for 5 min. The supernatant was discarded, and the bacterial pellet was resuspended in 1 mL of double-distilled autoclaved water (ddH_2_O). The washing step was repeated twice to ensure complete removal of residual TSB. After the final wash, the pellet was resuspended in 1 mL of ddH_2_O. A 100 µL aliquot of the bacterial suspension was diluted in 900 µL of ddH_2_O and subjected to optical density (OD) measurement at 600 nm using a spectrophotometer (Molecular Devices, LLC, San Jose, CA, USA) to determine bacterial concentration. Based on the OD value, the concentration was estimated to be approximately 2 × 10⁸ cells/mL, which was used as the initial stock for further serial dilutions.

Especially for the experiments involving multiple species or strains, *Salmonella enterica* subsp. *arizonae* ATCC 13314 (SA13314), *Escherichia coli* ATCC 25922 (*E. coli*), and *Listeria monocytogenes* ATCC 19111 (*L. mono*) were selected besides SE1045 cells. The bacterial colony recovery and culture followed the above description. To accurately control the spiked number of bacterial cells and calculate the recovery efficiency, the initial bacterial concentration was determined by plate counting. Serial dilutions of the washed bacterial suspension were prepared in ddH_2_O, and 100 µL aliquots of appropriate dilutions were spread onto TSA plates in triplicate. Plates were incubated at 37°C overnight, and colonies were enumerated to calculate the starting bacterial concentration based on dilution factors and plated volumes.

### Development of a smartphone application for data collection and processing

A customized smartphone application was developed to streamline image acquisition, processing, and data analysis, enabling users to efficiently manage the data processing workflow with minimal complexity. The application integrated a robust image processing pipeline via a WebView that interfaces with a WebAssembly-based progressive web application called ImJoy (52), a scientific image analysis tool optimized for mobile environments and inspired by the widely used ImageJ software (53). A custom JavaScript script embedded within the WebView facilitated advanced functionalities, including the execution of ImageJ macros, user-defined commands, and seamless transfer of processed images and data to the phone’s local storage.

Upon connecting a smartphone to a microscope via Wi-Fi, the application enabled real-time image capture and organization. Using the User Datagram Protocol, JPEG images were streamed directly to mobile devices, allowing users to organize images into structured folders. These images were then processed using ImJoy, which implemented a series of customizable image processing functions, including 8-bit conversion, threshold masking, bandpass filtering, and particle analysis to quantify the number of detected particles in each image. The extracted data were stored in easily accessible JSON files for further analysis.

To enhance usability and productivity, the application featured an intuitive user interface, allowing scans to be efficiently grouped into folders, shared with collaborators, and batch-processed, making it well-suited for large-scale studies. A detailed user interface and workflow were provided in the Supporting Information (Fig. S1).

### Protocol of the smartphone-based optical detection method

The preparation of the 3-MPBA-coated gold chips followed the procedure described in references 35, 36. Gold-coated slides (Thermo Scientific, Portsmouth, NH, USA) were cut into 0.5 × 0.5 cm² squares and placed in a 96-well plate. Each square was washed with 200 μL ethanol, air-dried, and coated with 200 μL of 40 mmol/L 3-MPBA. Chips were incubated at room temperature with shaking (50 rpm, 17 h), rinsed twice with 200 μL ddH_2_O, air-dried, and mounted on glass microscope slides with double-sided tape.

Stainless-steel 304 surfaces or HDPE (10 × 10 cm²) were cleaned with ddH_2_O, sterilized with 70% ethanol, and air-dried. Fifty microliters of diluted SE1045 suspension was applied (~25 evenly spaced droplets) and air-dried (5–10 min, room temperature). The surface was sampled with ethanol-premoistened Puritan 6” Sterile Standard Foam Swabs (Puritan Medical Company, Guilford, ME, USA) using a zigzag pattern with perpendicular overlap. The swab was placed in 300 μL ethanol within a 1 mL centrifuge tube, agitated 15 times, and pressed against the tube wall to recover liquid.

Five microliters of the recovered suspension was deposited on a 3-MPBA-coated chip and air-dried (20 s). To remove uncaptured particles, 1 mL ethanol was flushed over the chip, followed by another 20 s drying. Chips were imaged under a wireless digital smartphone microscope 50×–1,000× fixed focus, LED illumination; iWOBAC, (Amazon, USA) connected via Wi-Fi to an iPhone 11 (iOS 16.0 or later). Microscope brightness and focus were adjusted to obtain high-contrast images at a lens-to-chip distance of around 1.8 cm, producing a field of view of 2.0 × 1.2 mm^2^. Images were captured using a custom smartphone application, which was described in “Development of a smartphone application for data collection and processing,” and analyzed within 10 s. To standardize image processing, representative positive (bacteria present) and negative (no bacteria) images were first evaluated using ImageJ on a PC. Thresholding was optimized to select only uniform, dot-like particles while excluding irregular or aggregated structures (Fig. S9). The following parameters were adopted as defaults in the app’s ImageJ-based module: threshold 145–255; particle size 3–60 pixels; bandpass 0–5 pixels. The app then displayed bacterial counts directly on the smartphone screen.

In addition to the smartphone microscope system used throughout this study, two commercially available smartphone microscopes were evaluated for hardware comparison: a 40×–1,000× fixed-focus microscope with LED illumination (Jiusion; Amazon, USA) and a 50×–1,000× fixed-focus microscope with LED illumination (BEBANG; Walmart, USA). Identical SE1045 samples captured on 3-MPBA-coated gold chips were imaged using each device under illumination and magnification settings that provided clear visualization of individual particle features. The effective field-of-view areas of the Jiusion and BEBANG devices were measured as 2.3 × 1.2 mm and 2.8 × 1.5 mm, respectively. Because only a magnification range was indicated on the devices and the exact magnification used could not be directly determined, image analysis parameters were adjusted based on the observed scale bars. To account for differences in apparent particle size resulting from variations in imaging scale, particle size thresholds were optimized to include small, round features corresponding to bacterial cells. Specifically, particle sizes of 2–50 pixels were used for images acquired with the Jiusion device, and 1–30 pixels were used for images acquired with the BEBANG device, while all other image analysis parameters were kept constant.

### Recovery efficiency determination

Following bacterial cell preparation, the concentrations of four bacterial suspensions—SE1045, SA13314, *E. coli*, and *L. mono*—were determined by plate counting and adjusted to approximately 10⁷ cells/mL. A 50 µL aliquot of each diluted suspension was deposited onto a 10 × 10 cm² stainless-steel surface, resulting in surface-spiked bacterial loads of (1.37 ± 0.21) × 10⁶ CFU (SE1045), (1.60 ± 0.57) × 10⁶ CFU (SA13314), (1.16 ± 0.36) × 10⁶ CFU (*E. coli*), and (2.26 ± 0.02) × 10⁶ CFU (*L. mono*). After swab sampling, bacterial cells were released into 300 µL of release solution; however, due to liquid retention by the swab, the effective recovered volume was 200 µL. A 5 µL aliquot of the released solution was applied to a 3-MPBA-coated gold chip (5 × 5 mm²), and particle counting was performed on a defined imaging area of 2.0 × 1.2 mm². Particle counts obtained from optical images were recorded and used to back-calculate the total number of detected cells. Recovery efficiency was calculated by comparing the back-calculated detected cell number with the corresponding surface-spiked bacterial load using the following equation:


$$Recovery\;efficiency(\%)=\frac{(N_{particle\;counts\;on\;image}-N_{blank})\times 40\times 10.42}{N_{spiked\;bacterial\;number}}\times 100$$

where ${\text{N}}_{\text{parti}\text{cle counts on image}}$ is the particle count on sample images, ${\text{N}}_{\text{blank}}$ is the mean particle count from negative control images (swabbed surface with no bacterial loading), ${\text{N}}_{\text{spiked bacterial number}}$ is the surface-spiked bacterial number determined by plate counting, 40 represents the volumetric correction factor (200 µL total release volume divided by 5 µL imaged volume), and 10.42 represents the area correction factor (total chip area divided by the imaged area).

### ATP measurement

The Hygiena SystemSURE Plus ATP Monitoring System (with UltraSnap surface ATP test swab) (Hygiena LLC, Camarillo, CA, USA) was commercially available and used in this study. The bacterial suspension was applied to 10 × 10 cm² stainless-steel surfaces following the procedure described in “Protocol of the smartphone-based optical detection method.” Surface sampling using ATP test swabs, activation of the ATP monitoring meter, and measurement of RLU were conducted according to the manufacturer’s instructions.

### Bacterial detection with the presence of food matrices

All foods and beverages used in this study were commercially available, which included corn oil, starch, spinach, orange juice, egg, whole milk, peanut butter, soy protein, and wheat protein. The liquid samples (corn oil, orange juice, and whole milk) were diluted with ddH_2_O to the concentration of 1% (vol/vol). In terms of rest solid foods, 1 gram of each food was weighed and homogenized in 100 mL ddH_2_O with a blender for 1 min, resulting in 1% (wt/vol) food matrices samples.

To assess the influence of food matrices on background signals in both the smartphone-based optical detection method and the ATP swab tests, 50 μL of each 1% food matrix suspension was distributed onto stainless-steel surfaces. The detection procedures were then carried out according to “Protocol of the smartphone-based optical detection method” or “ATP measurement.” The ddH_2_O without food matrices served as the negative control.

To evaluate the effect of food matrices on bacterial detection performance, a spike-recovery approach was used. Specifically, 1 mL of each 1% food matrix suspension was used to resuspend the washed bacterial pellet (as described in “Bacterial cell preparation”). The bacterial suspension was then subjected to 10-fold serial dilution in the 1% food matrix suspension, yielding a final concentration of 2 × 10⁸ cells/mL. Fifty microliters of the mixture was applied to stainless-steel surfaces and analyzed using the protocols in “Protocol of the smartphone-based optical detection method” or “ATP measurement.” A bacterial suspension of 2 × 10⁸ cells/mL in ddH_2_O served as the control. To eliminate the influence of food matrices on background readings, the actual detected bacterial results were calculated using the following equation:


$${\text{R}}_{\text{bacteria}}\text{=}{\text{R}}_{\text{(1\textbackslash{}\% food matrices}\text{\textbackslash{} }\text{+}\text{\textbackslash{} }\text{bacterial cells)}}\text{-}{\text{R}}_{\text{(1\textbackslash{}\% food matrices)}}$$

where ${\text{R}}_{\text{bacteria}}$ represents the actual detected bacterial results; ${\text{R}}_{\text{(1\textbackslash{}\% food matrices}\text{\textbackslash{} }\text{+}\text{\textbackslash{} }\text{bacterial cells)}}$ represents the detection results from the surfaces containing both bacteria and food matrices; ${\text{R}}_{\text{(1\% food matrices)}}$ represents the detection results from the surfaces only containing food matrices.

The percent bias (%Bias) was calculated as the following equation:


$$\%Bias\text{=}\frac{{\text{R}}_{\text{F}\text{ood matrices}}\text{-}{\text{R}}_{\text{W}\text{ater}}}{{\text{R}}_{\text{Water}}}\times 100$$

where ${\text{R}}_{\text{Fo}\text{od matrices}}$ represents actual detected bacterial results under the given food matrices, and ${\text{R}}_{\text{Water}}$ represents actual detected bacterial results under the water control.

### Evaluation of shelf life of 3-MPBA-coated gold chip

3-MPBA-coated gold chips were prepared as described in “Protocol of the smartphone-based optical detection method” and stored in covered Petri dishes at room temperature (26°C) for 0 days, 1 week, 2 weeks, 1 month, 2 months, 4 months, and 6 months. At each time point, chips were retrieved and evaluated for (i) background signal and (ii) bacterial capture capability. For background assessment, chips were directly imaged under the smartphone microscope, and background particles were counted. For bacterial capture, 5 μL of an SE1045-ethanol suspension (2 × 10⁸ cells/mL) was applied to each chip, air-dried, washed with 1 mL ethanol, and re-dried. Chips were then imaged as described in “Protocol of the smartphone-based optical detection method,” and captured bacterial cells were quantified. Results were compared across storage durations to evaluate chip stability over time.

### Application of smartphone-based optical detection for bacteria on a public water fountain surface

To assess real-world applicability, a stainless-steel public water fountain (Chenoweth Lab, UMass) was sampled at the end of the day when the surface was visibly dry. The fountain was not routinely sanitized, allowing natural bacterial accumulation and potential early-stage biofilm development. Because the fountain panel could not accommodate a standard 10 × 10 cm² swabbing area, a custom 5 × 10 cm² frame was used. Two adjacent areas were designated: one for smartphone-based optical detection and the other for culture-based plate counting.

For optical detection, the swabbing and sample preparation were conducted following the procedure described in “Protocol of the smartphone-based optical detection method.” Briefly, swab releasing solution was deposited onto 3-MPBA-coated gold chips in triplicate, imaged using a smartphone microscope, and processed with the standardized particle analysis parameters. Average particle counts per image were obtained and converted to calculated bacterial load using the calibration curve in Fig. 3c.

To back-calculate bacterial number from plate counting results, a standard curve was generated by depositing 50 μL of known concentrations (2 × 10⁸ to 2 × 10¹ cells/mL) of SE1045 cells on stainless-steel surfaces (10 × 10 cm²). A sterile Puritan 6” foam swab pre-moistened with ddH_2_O was used to swab the area with a consistent zigzag motion. The swab was eluted into 300 μL sterile saline, agitated, and pressed against the tube wall before discarding. Serial 10-fold dilutions were performed, and 100 μL of each dilution was plated on TSA in triplicate. Plates were incubated at 37°C for 72 h, and CFU were counted. Final bacterial loads were calculated based on dilution factors and recovered volume and plotted against the known inoculated cell numbers to generate a linear regression standard curve (Fig. S7). For the water fountain surface sample, the same swabbing and plating protocol was applied. Bacterial colonies were quantified and converted to calculated bacterial numbers using the established standard curve. This allowed for direct comparison between optical and culture-based quantification methods under field conditions.

### Statistical analysis

To ensure reliable and accurate results, each gold chip was imaged in three to four different regions, and the particle count results were averaged to yield a single replicate result. Each sample was conducted with three technical replicates, and the results were calculated as mean and SD.

The LOD was calculated using the equation below:


LOD=Mean of blank+3×SD of blank

where the blank represents negative control samples obtained by swabbing surfaces without bacterial loading, followed by release of the swab into extraction solution and application of the released liquid onto 3-MPBA-coated gold chips.

Statistical significance was assessed using paired *t*-tests. Significance levels were denoted as follows: *P* < 0.05, *P* < 0.01, *P* < 0.001, and n.s. for not significant (*P* > 0.05). All statistical analyses were performed using OriginLab 2021, and the *P*-values for individual comparisons are reported in the main text or figure legends.

## Data Availability

All data generated or analyzed during this study are included in this article and its supplemental material. Image processing was performed in the mobile application using the open-source imageJ.js platform (https://github.com/imjoy-team/imagej.js).

## SUPPLEMENTAL MATERIAL

Supplemental material aem.00073-26-s0001.docx Fig. S1 to S9; Tables S1 and S2.

ASM does not own the copyrights to Supplemental Material that may be linked to, or accessed through, an article. The authors have granted ASM a non-exclusive, world-wide license to publish the Supplemental Material files. Please contact the corresponding author directly for reuse.
