# Supplementary material for: A smartphone-based optical detection for rapid and reliable quantification of bacterial contamination on stainless-steel surfaces
Source: Appl Environ Microbiol. 2026 Apr 13;92(5):e00073-26. doi: 10.1128/aem.00073-26 (PMC13188859; doi:10.1128/aem.00073-26)
Supplement: Supplemental material — Fig. S1 to S9; Tables S1 and S2. [file aem.00073-26-s0001.docx]

**Supporting information**

**A Smartphone-Based Optical Detection for Rapid and Reliable Quantification of Bacterial Contamination on Stainless-Steel Surfaces**

**Authors:** Yuzhen Zhang^a^, Suraj Pathak^b^, Gabriella Curry^c^, Ngoc Vu^d^, Zili Gao^a^, and Lili He^a,d*^

**Affiliations:**

^a^Department of Food Science, University of Massachusetts, Amherst, MA 01003, USA.

^b^Department of Computer Science, University of Massachusetts, Amherst, MA 01003, USA.

^c^Department of Chemical Engineering, University of Massachusetts, Amherst, MA 01003, USA.

^d^Department of Chemistry, University of Massachusetts, Amherst, MA 01003, USA.

* Corresponding author:

Dr. Lili He, E-mail: [lilihe@umass.edu](mailto:lilihe@umass.edu); Fax: +1 413 545 1262; Tel: +1 413 545 5847


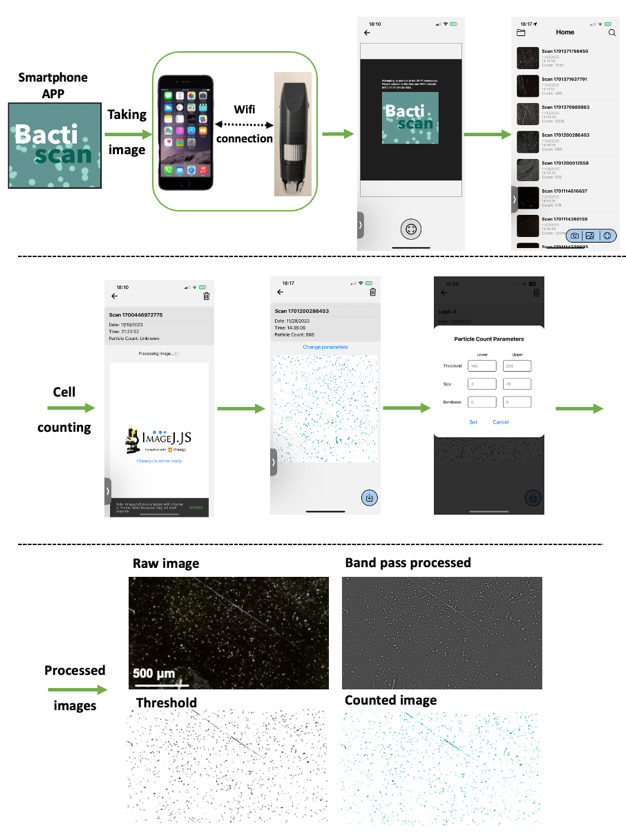


FigS1. The workflow of image collection and processing uses the customized smartphone app embedded with the ImageJ package. The scale bar represented 500 μm length.


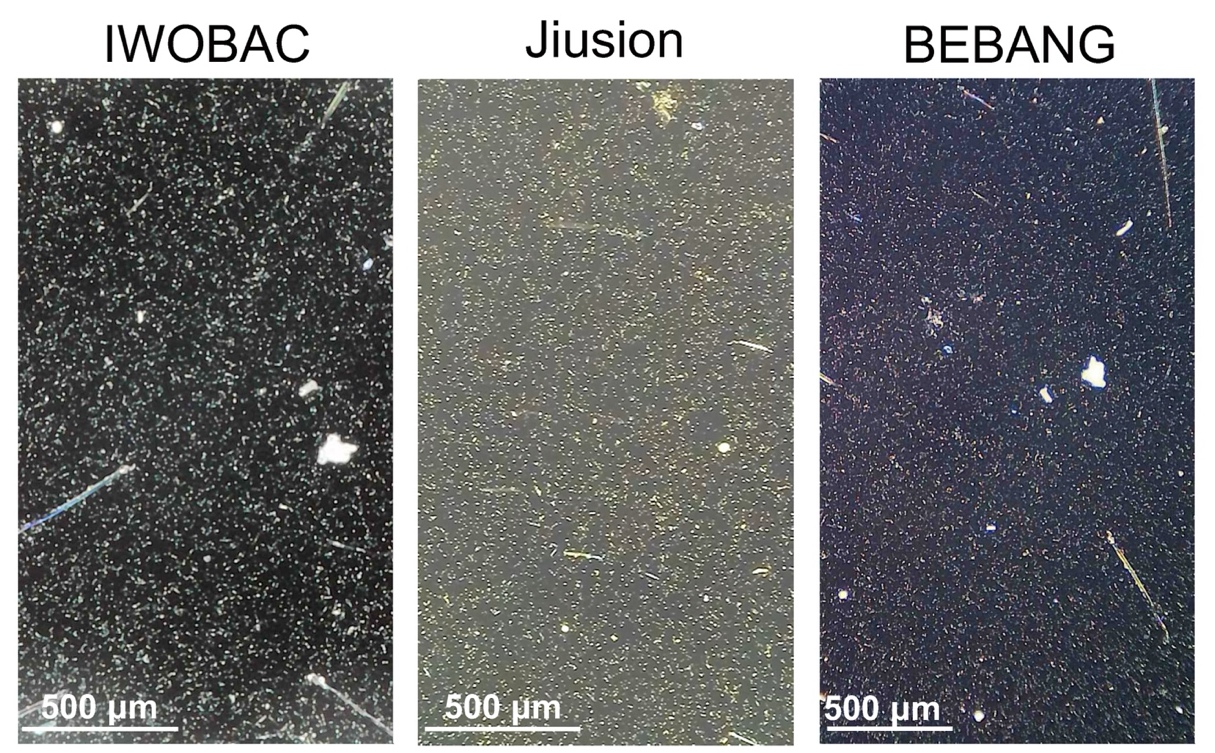


FigS2. Optical images of SE1045 captured on 3-MPBA-coated gold chips using three commercially available smartphone-based microscope systems (IWOBAC, Jiusion, and BEBANG). SE1045 cells were recovered by swabbing stainless-steel surfaces inoculated at 10⁸ cells. Representative fields of view are shown. Scale bars represent 500 µm.

Table S1. Comparison of particle counts obtained from SE1045 cells captured on 3-MPBA-coated gold chips using three commercially available smartphone microscope systems. Reported values represent mean ± standard deviation from replicate images collected. Particle counts were normalized to counts per mm² based on the effective field-of-view area of each device. %CV indicates the coefficient of variation.

| Product brand | View area (mm*mm) | Particle counts on image | Counts per mm^2^ | %CV |
| --- | --- | --- | --- | --- |
| IWOBAC | 2*1.2 | 3112 ± 236.87 | 1296.67 ± 98.69 | 9.20 |
| Jiusion | 2.3*1.2 | 4299.67 ± 248.66 | 1557.85 ± 90.09 |  |
| BEBANG | 2.8*1.5 | 5937.67 ± 119.50 | 1413.73 ± 28.45 |  |


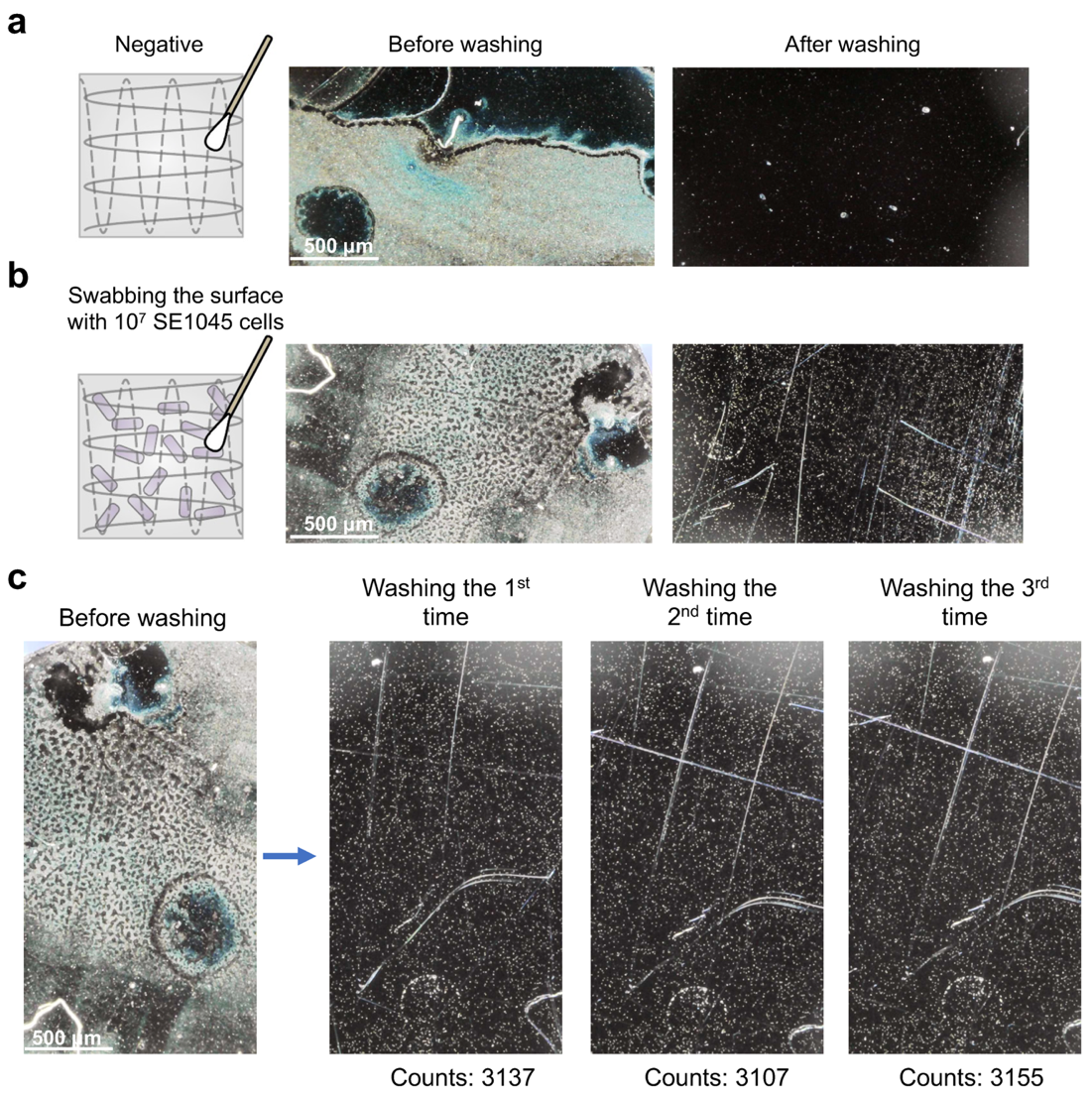


FigS3. Washing steps in the smartphone-based method for bacterial detection. Optical images (smartphone-based microscope) of 5 μL of suspension recovered from the surface (a) with nothing dropped on 3-MPBA-coated gold chips and dried, without and with a washing step; (b) with 10^7^ SE1045 cells; (c) Optical images (smartphone-based microscope) of 5 μL of bacterial suspension recovered from the surface with 10^7^ SE1045 cells dropped on the 3-MPBA-coated gold chip to dry, then washed 0, 1, 2, and 3 times, respectively. The scale bar represented 500 μm length.


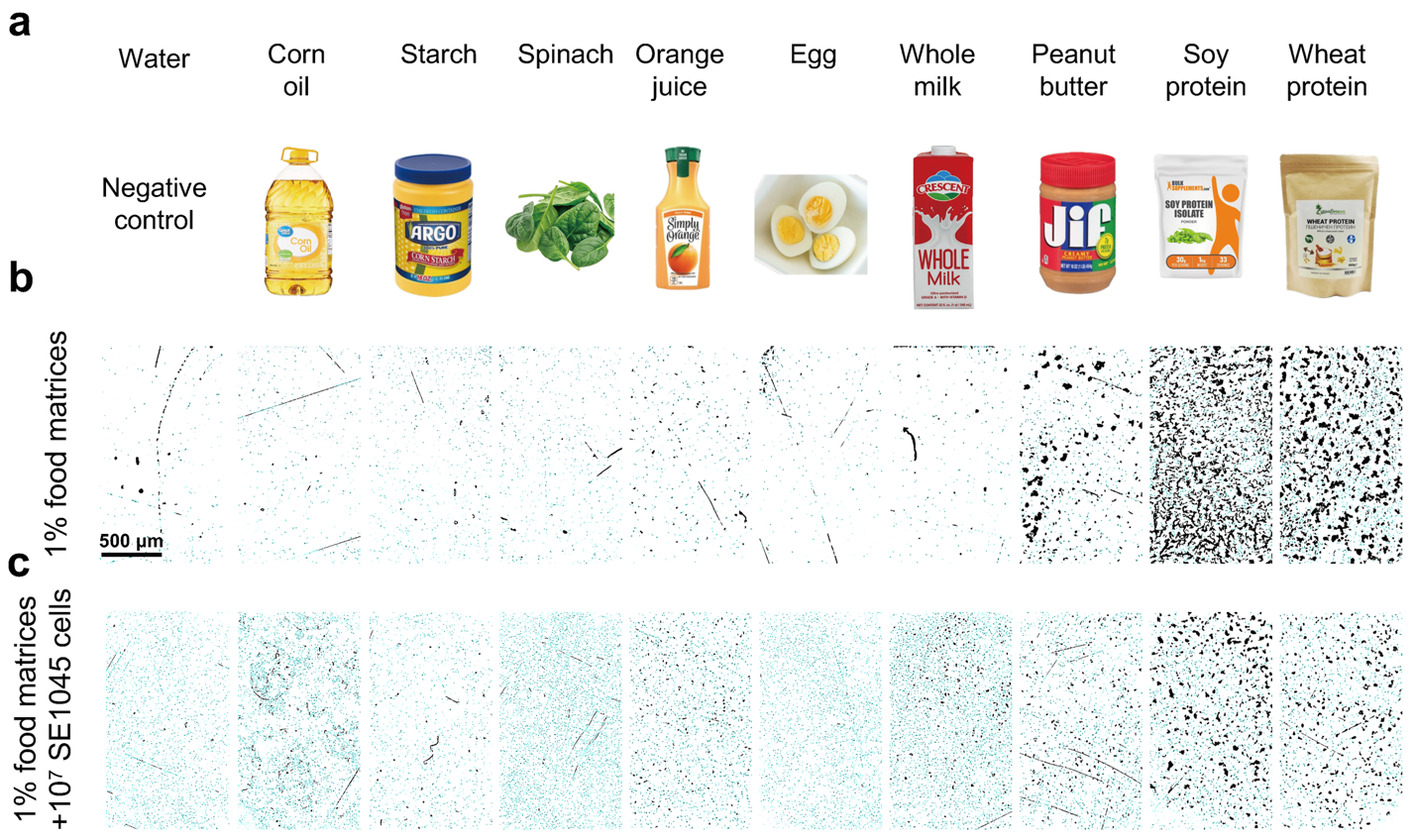


FigS4. Counted images of food matrices, and food matrices with bacteria recovered from surfaces using the smartphone-based method. (a) Pictures of food matrices that were introduced to investigate the influence of the smartphone-based method; (b) Counted images of samples recovered from surfaces with 1% different food matrices on 3-MPBA-coated gold chips; (c) Counted images of samples recovered from surfaces with 1% different food matrices and 10^7^ SE1045 cells on 3-MPBA-coated gold chips. The scale bar represented 500 μm length.


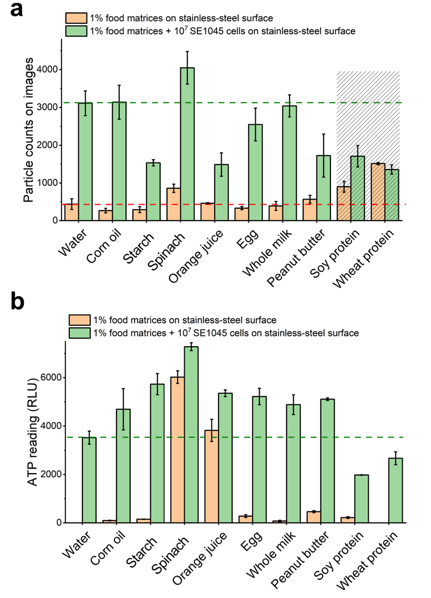


FigS5. Bar graphs of results of food matrices and food matrices with bacteria recovered from surfaces using the smartphone-based method and ATP swab kit. (a) The particle counts on images from Fig. S3 (b) (orange bar) and (c) (green bar). The diagonal pattern that covered the results from soy and wheat protein represented the inaccurate calculated results due to the big trunks on images; (b) The ATP reading results of samples recovered from the surfaces only with 1% different food matrices (orange bar) and 1% different food matrices and 10^7^ SE1045 cells (green bar). The orange and green dashed line was drawn based on the average value of the orange and green bar under water, respectively. Data were represented as mean ± SD of three technical replicates (n = 3).

## **Table S2.** Quantitative comparison of matrix effects on smartphone-based optical detection and ATP monitoring. All values are reported as mean ± SD. Percent bias (%Bias) and coefficient of variation (%CV) were calculated relative to the water control for each method.

| Food matrix | **Smartphone-based optical detection** | | | **Hygiena™ UltraSnap™ Surface ATP monitoring system** | | |
| --- | --- | --- | --- | --- | --- | --- |
|  | Mean ± SD  (particle counts) | %Bias | %CV | Mean ± SD  (ATP readings) | %Bias | %CV |
| **Water (control)** | 2670.50 ± 336.15 | - | - | 3521.67 ± 270.54 | - | - |
| **Corn oil** | 2868.00 ± 464.27 | 7.40 | 5.04 | 4599.67 ± 836.40 | 45.80 | 18.77 |
| **Starch** | 1240.75 ± 95.10 | −53.54 | 51.70 | 5579.33 ± 441.73 | 59.03 | 31.97 |
| **Green leaf** | 3186.50 ± 440.83 | 19.32 | 12.46 | 1257.00 ± 195.81 | −69.70 | 67.02 |
| **Orange juice** | 1140.00 ± 533.12 | −57.31 | 56.80 | 1534.00 ± 334.34 | −64.82 | 55.60 |
| **Egg** | 2212.00 ± 449.47 | −17.17 | 13.28 | 4941.67 ± 297.21 | 38.87 | 23.73 |
| **Whole milk** | 2647.00 ± 296.28 | −0.88 | 0.62 | 4811.00 ± 395.66 | 37.92 | 21.88 |
| **Peanut butter** | 1154.75 ± 610.33 | −56.76 | 56.04 | 4645.33 ± 86.80 | 20.24 | 19.46 |
| **Soy protein** | 808.00 ± 403.90 | −69.74 | 75.72 | 1759.33 ± 27.39 | −54.28 | 47.19 |
| **Wheat protein** | −190.75 ± 76.45 | −107.14 | 163.18 | 2670.67 ± 264.64 | −23.06 | 19.44 |


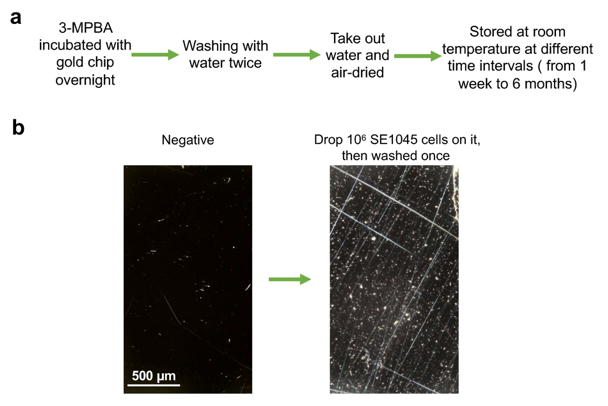


FigS6. The protocol for evaluating the shelf life of the 3-MPBA-coated gold chips. (a) The workflow of manufacturing and storing 3-MPBA-coated gold chips; (b) Optical images (smartphone-based microscope) of the 3-MPBA-coated gold chips stored under different days (negative control), then dropped with 10^6^ SE1045 cells and then washed once with 1 mL flushing ethanol. The scale bar represented 500 μm length.


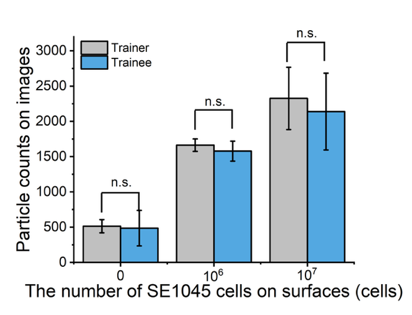


FigS7. The comparison of particle counts on images of the samples recovered from the surfaces loading 0, 10^6^, and 10^7^ cells by the trainer (gray bar) and trainee (blue bar). Statistically significant differences were determined by paired t-test, n.s. illustrated no significant difference level (P > 0.05). Data are represented as mean ± SD of three technical replicates (n = 3).


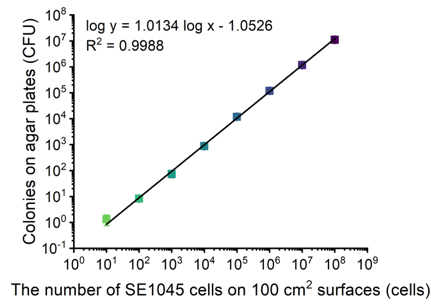


FigS8. Calibration curve correlating the number of SE1045 colonies recovered from stainless-steel surfaces via swabbing with the actual number of SE1045 cells present on the surfaces. Data were represented as mean ± SD of three technical replicates (n = 3).


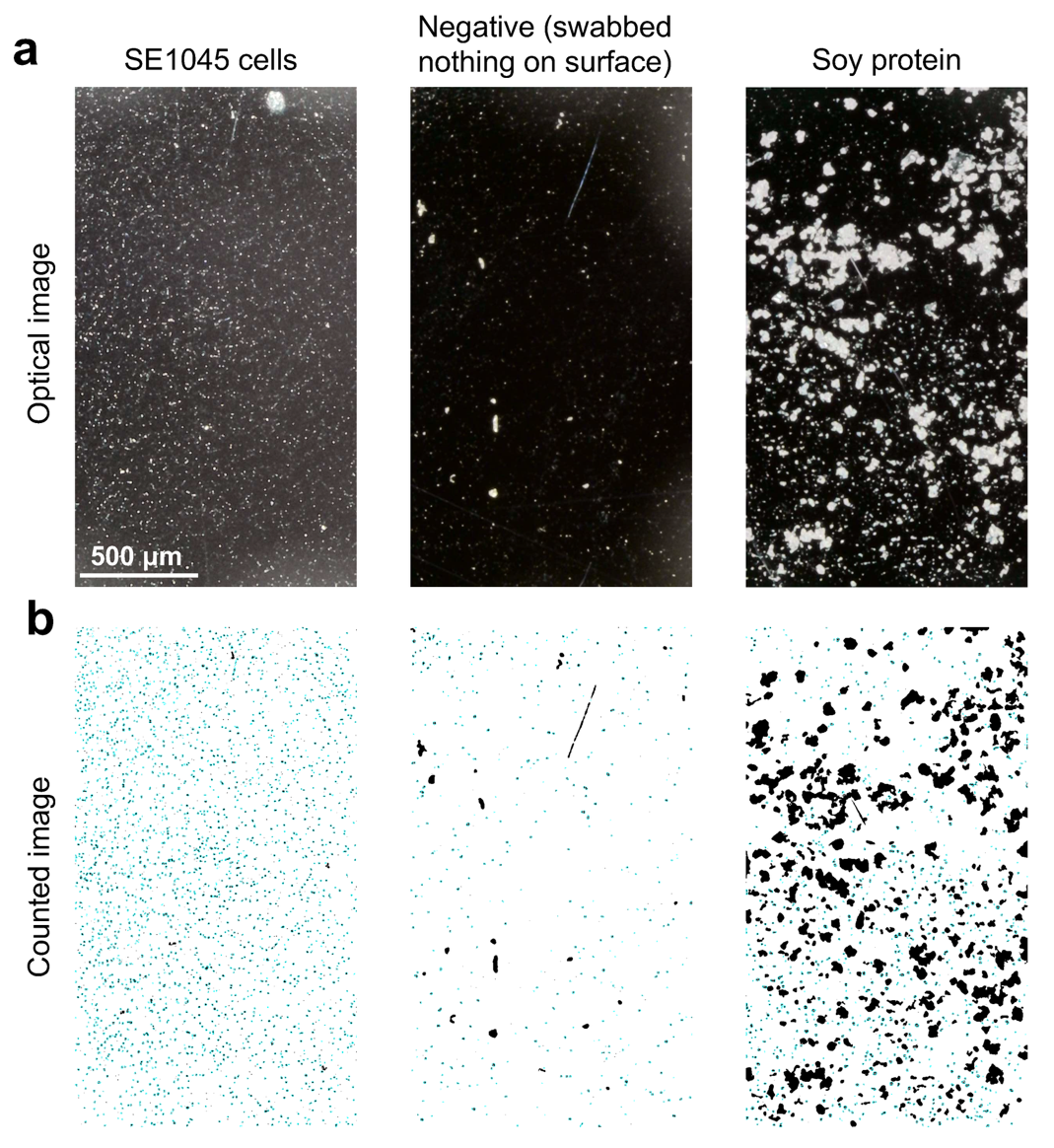


FigS9. Images of SE1045 bacterial cells, negative control (no sample on surface), and soy protein background captured on 3-MPBA-coated gold chips. (a) Optical images acquired using a smartphone-based microscope; (b) Corresponding counted images processed using ImageJ within the customized app, with threshold settings of 145–255, particle size range of 3–60 pixels, and bandpass filter set to 0–5. The particles with blue solid line marked were counted while others were excluded out. The scale bar represented 500 μm length.
